# Bioactive Limonene-Derived Oligomer in Gelatin Hydrogels: Impact of Cross-Linking Chemistry on Physicochemical Properties and Wound Healing Performance

**DOI:** 10.1021/acsami.5c20409

**Published:** 2025-12-24

**Authors:** Roniérik Pioli Vieira, Guilherme Frey Schutz, Laurens Parmentier, Oana-Maria Chirliu, Aurelian-Sorin Pasca, Lenuta Profire, Sandra Van Vlierberghe

**Affiliations:** † 28132 Universidade Estadual de Campinas (UNICAMP), School of Chemical Engineering (FEQ), Albert Einstein Avenue, 500, 13083-852 Campinas, São Paulo, Brazil; ‡ Polymer Chemistry and Biomaterials Group (PBM), Centre of Macromolecular Chemistry (CMaC), Department of Organic and Macromolecular Chemistry, Faculty of Sciences, Ghent University, Krijgslaan 281, S4, 9000 Ghent, Belgium; § Department of Pharmaceutical and Therapeutic Chemistry, Faculty of Pharmacy, “Grigore T. Popa” University of Medicine and Pharmacy of Iaşi, 16 University Street, 700115 Iaşi, Romania; ∥ Faculty of Veterinary Medicine, “Ion Ionescu de la Brad” University of Life Sciences, 8 Mihail Sadoveanu Alley, 700489 Iaşi, Romania

**Keywords:** antioxidant, ROS, wound dressing, limonene, orange essential oil, biopolymer

## Abstract

Antioxidant photo-cross-linkable hydrogels have garnered significant interest for biomedical applications, but challenges such as additive thermal instability and interference with cross-linking remain. Here, we report the first systematic comparison of a limonene-derived oligomer (PLM) incorporated into two distinct photo-cross-linkable gelatin platforms, highlighting how cross-linking chemistry governs antioxidant performance and network integrity. PLM was incorporated at 5% and 10% (w/w, relative to gelatin), comparing the performance of gelatin-methacryloyl (GelMA) and gelatin-norbornene (GelNB) hydrogels in terms of cross-linking efficiency, antioxidant retention, release profile, and biocompatibility upon PLM incorporation. While PLM negatively affected the physicochemical properties of GelMA, its incorporation into GelNB did not show similar drawbacks. High-resolution magic angle spinning (HR-MAS) ^1^H NMR spectra revealed a significant drop in double bond consumption (DC) for GelMA, from 71% to 46% in GelMA/PLM10. In contrast, GelNB demonstrated nearly complete DC (98%) even in GelNB/PLM10, indicating efficient cross-linking despite the presence of the antioxidant. Release profiles suggested a Fickian diffusion mechanism, with higher diffusivity values for GelNB, further highlighting differences in the matrix networks. Additionally, while PLM increased GelMA’s antioxidant capacity from 6% to 19%, GelNB with PLM showed an impressive enhancement, reaching up to 90%. Both GelMA and GelNB hydrogels with 5% (w/w) PLM supported healthy cell morphology and viability above 85% over 7 days. *In-vivo*, GelNB/PLM5 demonstrated excellent biocompatibility over 18 days, with no significant inflammation compared to controls. Notably, this sample achieved the highest wound closure (94.90%) by day 18, outperforming the positive control (Sorbalgon). Overall, the results demonstrate that the thiol–ene cross-linking pathway enables superior preservation of antioxidant functionality and network integrity, positioning GelNB/PLM5 as a promising candidate for advanced wound-healing applications.

## Graphical abstract

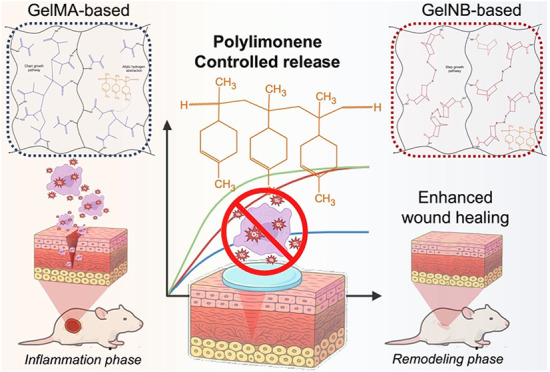

## 1 Introduction

Reactive oxygen species (ROS) play an important role in wound healing. However, their overproduction can result in delayed healing, particularly in chronic wounds.^1,2^ Excessive ROS can lead to oxidative stress, damaging surrounding tissues and hindering angiogenesis, collagen synthesis, and epithelialization.^1,3–6^ The imbalance between ROS production and antioxidant defenses contributes to prolonged inflammation and delayed transition to the proliferative phase of healing.^7^ As an example, diabetic wounds exhibit sustained oxidative stress characterized by increased levels of ROS, which exacerbates inflammation and impedes tissue regeneration and angiogenesis.^8^ Thus, there is a consensus in literature that effective management of ROS levels is critical in promoting wound healing.

Strategies to reduce ROS accumulation, particularly through antioxidant treatments, are widely explored as potential interventions to improve wound healing outcomes.^9–11^ Among these approaches, antioxidant hydrogels have shown promising results in mitigating oxidative stress and accelerating healing.^12–14^ A notable example is the incorporation of essential oils into gelatin-based hydrogels, combining the benefits of gelatin’s biocompatibility with the therapeutic properties of essential oils.^15,16^ Gelatin (Gel), a natural polymer derived from collagen, provides an optimal environment for cell adhesion and proliferation while offering excellent moisture retention, which is an essential factor in wound healing.^17–19^ When Gel is combined with different essential oils, the resulting hydrogels not only maintain hydration but also introduce strong antioxidant properties,^20,21^ further supporting tissue regeneration.

However, the incorporation of antioxidants into wound dressings poses significant challenges that can limit their efficacy. One major concern is the volatility of many antioxidant agents, particularly natural compounds such as essential oils and plant-derived phenols, which can lead to substantial losses during storage and application.^22,23^ These compounds may also degrade or evaporate under common sterilization procedures, such as γ irradiation,^24^ ethylene oxide treatment,^25,26^ or autoclaving,^27^ compromising their functional activity before clinical use. Furthermore, the incorporation of antioxidant agents into photo-cross-linkable hydrogels to improve their physicochemical properties may interfere with the photopolymerization process. This interference is often due to the radical-scavenging nature of antioxidants,^11,28^ which quench free radicals necessary for network formation, resulting in incomplete cross-linking and poor gel stability.^29,30^

More importantly, the presence of antioxidants not only compromises the efficiency of polymer network formation but may also result in chemical changes to the antioxidant itself during photo-cross-linking, potentially leading to a partial or complete loss of its bioactivity.^31,32^ As a consequence, the intended mitigation of oxidative stress by these additives can be significantly diminished in the final biomaterial. Although post-cross-linking loading of antioxidants into hydrogels offers a potential strategy to preserve their activity, it introduces its own challenges, such as limited diffusion into dense polymer networks,^33^ poor long-term retention,^34^ and difficulty in achieving uniform distribution.^35,36^ These limitations highlight the need for innovative formulations to effectively balance antioxidant functionality with the structural, bioactive, and mechanical requirements of advanced wound dressings.

In this study, we present the first comprehensive evaluation of the effects of a limonene-derived oligomeric antioxidant additive on the photo-cross-linking behavior and physicochemical properties of two biomedically relevant hydrogel systems: gelatin methacryloyl (GelMA) and gelatin norbornene (GelNB). GelMA is known for its free-radical-based photo-cross-linking,^18^ while GelNB typically relies on thiol–ene chemistry in the presence of multifunctional thiols.^37^ An additional contribution of this study is the introduction of polylimonene (PLM) as a highly stable antioxidant additive for the development of biomaterials. This oligomer holds significant potential for the valorization of citrus byproducts, as limonene constitutes over 90% of citrus peel essential oil.^38,39^ While PLM has been previously investigated as an additive in food packaging applications,^40–42^ its biomedical potential remains scarcely explored, with no existing studies evaluating its impact on *in vivo* models for biocompatibility assessment. Importantly, unlike conventional small-molecule antioxidants that often volatilize, degrade, or quench radicals essential for network formation, PLM’s oligomeric structure provides enhanced thermal and photochemical stability. In addition, we hypothesize that PLM may scavenge free radicals in GelMA, thereby interfering with chain-growth methacrylate polymerization, whereas its impact is substantially reduced in GelNB, where the thiol–ene step-growth mechanism is less sensitive to radical depletion. This dual behavior helps explain why PLM minimizes interference with photo-cross-linking reactions, particularly in thiol–ene systems.

Therefore, this research aimed to investigate how PLM influences photo-cross-linking efficiency in each system (GelMA versus GelNB) and whether it retains its antioxidant functionality after cross-linking. By elucidating these mechanistic differences, this work demonstrates how PLM overcomes the typical limitations of antioxidant incorporation and enables improved preservation of both network integrity and antioxidant activity. This fundamental study not only provides insight into the balance between structural integrity and bioactivity in antioxidant-loaded hydrogels but also is a bottom line for the design of other photo-cross-linkable biomaterials incorporating antioxidants. Additionally, PLM release profiles from both gelatin matrices were assessed, and concentration thresholds for cellular viability were identified. Finally, the most promising formulation was evaluated in a wound healing model, demonstrating the *in vivo* biocompatibility of PLM-loaded gelatin-based hydrogels for future biomedical applications. Figure 1 provides a schematic representation of the methodological approach adopted in this work.

**Figure 1:**
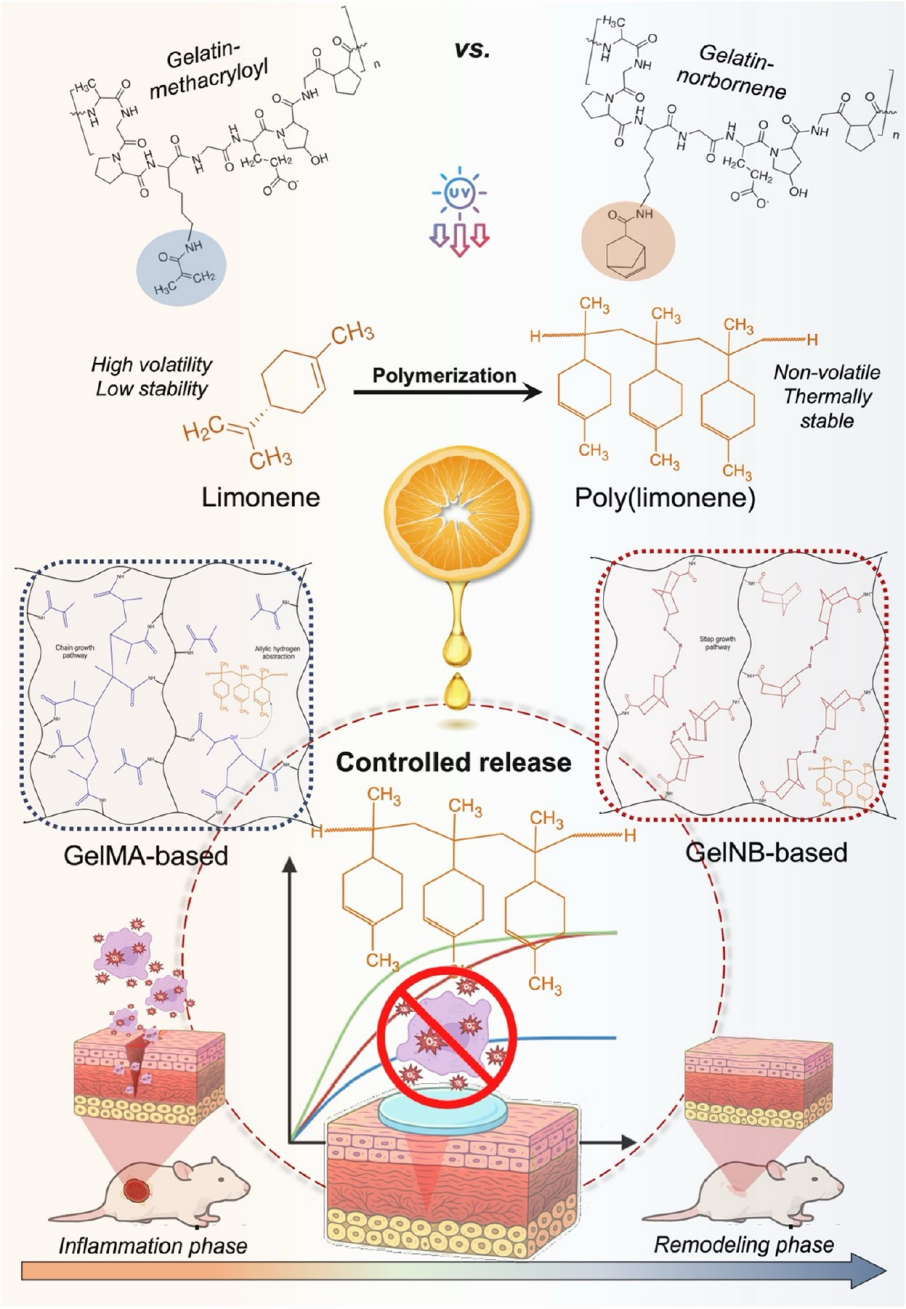
Schematic overview of the study design, illustrating the incorporation of the limonene-derived oligomer (PLM) into GelMA and GelNB precursor solutions, the distinct photo-cross-linking mechanisms involved (chain-growth vs thiol–ene), and the resulting effects on hydrogel physicochemical properties, antioxidant performance, and PLM release.

## 2 Materials and Methods

### 2.1 Chemicals

#### 2.1.1 Chemicals for Synthesis

Gelatin (Gel) type B (from bovine hides) with a number-average molecular weight (*M̅*_*n*_) of 66.9 kDa and dispersity (Đ) of 2.304 (size exclusion chromatogram as Supporting Information) was supplied by Rousselot (Ghent, Belgium). Methacrylic anhydride (MA, purity ≥94%), 2,2-diphenyl-1-picrylhydrazyl (DPPH, purity ≥99%), sodium hydroxide (NaOH, PA), predominantly endo 5-norbornene-2-carboxylic acid (NB, purity >98%), d,l-dithiothreitol (DTT, purity >99%), and dimethyl sulfoxide (DMSO, purity >99%), *N*-(3-(dimethylamino)­propyl)-*N*′-ethylcarbodiimide hydrochloride (EDC, purity >98%) was obtained at TCI (Zwijndrecht, Belgium). *N*-hydroxysuccinimide (NHS, purity >98%) was purchased at Acros (Geel, Belgium). Pluronic block copolymer based on poly­(ethylene oxide)-*b*-poly­(propylene oxide)-*b*-poly­(ethylene oxide) (PEO-*b*-PPO-*b*-PEO) (F127, 80% PEO, *M̅*_*n*_ 8400 Da) was supplied by BASF (Belgium). R-(+)-Limonene (LIM, purity >93%), *N*,*N*,*N*′,*N*″,*N*″-pentamethyldiethylenetriamine (PMDETA, purity ≥99%), dimethylacetamide (DMA, purity ≥99%), benzophenone (BP, purity ≥99%), and 2,2,2-tribromoethanol (TBE, purity ≥97%) were purchased from Sigma-Aldrich (Brazil), lithium (2,4,6-trimethylbenzoyl)­phenylphosphinate (Li-TPO-L or LAP) was synthesized according to a previously reported protocol.^43^ The dialysis membranes Spectra/Por were obtained from Polylab (Belgium).

#### 2.1.2 Chemicals for *In Vitro* and *In Vivo* Studies

Dulbecco’s Modified Eagle Medium (DMEM), fetal bovine serum (FBS), penicillin/streptomycin (P/S), calcein-acetoxymethyl/propidium iodide (Ca-AM/PI) were acquired from Sigma-Aldrich (Belgium). 3-(4,5-dimethylthiazol-2-yl)-5-(3-carboxymethoxyphenyl)-2-(4-sulfophenyl)-2H-tetrazolium (MTS) was supplied by Abcam. The chemicals for *in vivo* studies included isoflurane for anesthesia, potassium chloride (KCl) for intracardiac euthanasia, and 10% buffered formaldehyde as the fixative for tissue preservation. Histological processing involved paraffin for embedding, along with xylene and graded ethanol solutions for deparaffinization and rehydration. For staining, a Masson’s trichrome variant was employed, consisting of hematoxylin, eosin, and methylene blue.

### 2.2 Synthesis of Gelatin-Methacryloyl (GelMA)

Methacryloyl-modified gelatin (GelMA) was synthesized as previously described.^18^ Briefly, 100 g gelatin type B (38.5 mmol of NH_2_) was dissolved in 1 L phosphate buffer at 40 °C. The pH was adjusted to 7.8 with few drops of a 5 M NaOH solution. After that, 2.5 mol equivalents of methacrylic anhydride (MA) were added, i.e., 96.25 mmol (14.34 mL). The reaction occurred under stirring for 1 h at 40 °C [Figure S1­(a), **(1)**, Supporting Information], and then 1 L double distilled water was added. The solution was dialyzed (Spectra/Por 4 dialysis membranes, molar mass cut off 12000–14000 Da) using distilled water at 40 °C for 24 h, changing the water bath 5 times. After dialysis, the pH was adjusted to 7.4 using the 5 M NaOH solution. Purified GelMA solution was transferred to Petri dishes and frozen at −20 °C. Finally, the samples were freeze-dried for 48 h (−82 °C and 0.4 mbar). The GelMA degree of substitution (DS ≈ 95.6%) was calculated from the ^1^H NMR spectrum [Figure S2­(a), Supporting Information].

### 2.3 Synthesis of Gelatin-Norbornene (GelNB)

Norbornene-modified gelatin (GelNB) was synthesized as previously reported,^37,43^ considering 15 g of gelatin type B (5.78 mmol of NH_2_) as a calculation basis for each batch. First, 2.5 mol equivalents relative to the gelatin primary amines (i.e., 1.77 mL, ∼14.45 mmol) of 5-norbornene-2-carboxylic acid (NB) were dissolved in 75 mL of dried DMSO under an argon atmosphere. Then, 2 equiv of EDC (2.21 g, ∼11.56 mmol) were added, and the activation occurred for 5 min [Figure S1­(a), **(2)**, Supporting Information], followed by the addition of 3 equiv of NHS (1.99 g, ∼17.34 mmol) to obtain an activated succinimidyl ester via reaction for 25 h under an argon atmosphere [Figure S1­(a), **(3)**, Supporting Information]. In the final reaction step, gelatin type B (15 g) was dissolved in 450 mL DMSO at 50 °C and was mixed with the reaction system containing the activated succinimidyl ester. This mixture was allowed to react for another 12 h [Figure S1­(a), **(4)**, Supporting Information].

Next, the mixture was precipitated in a 10-fold excess of acetone, filtered on filter paper (pore size 12–15 μm) using a Büchner filter to remove the formed urea side products and DMSO, followed by dissolving in distilled water. The solution was dialyzed (Spectra/Por 4 dialysis membranes, molar mass cut off 12,000–14,000 Da) using distilled water at 40 °C for 24 h, changing the water bath 5 times. Then, the pH of the mixture was adjusted to 7.4 using 5 M NaOH. Purified GelNB solution was transferred to Petri dishes and frozen at −20 °C. Finally, the samples were freeze-dried for 48 h (−82 °C and 0.4 mbar). The GelNB degree of substitution (DS ≈ 84.8%) was calculated from the ^1^H NMR spectrum [Figure S2­(a), Supporting Information].

### 2.4 Synthesis of Polylimonene (PLM)

The limonene oligomer (PLM) was synthesized following a previously established method.^44^ Initially, LIM (monomer) and DMA (solvent) were combined in 20 mL glass vials in a 1:1 volume ratio. To this mixture, PMDETA (electron donor amine), TX (catalyst), and TBE (initiator) were added in a molar ratio of [LIM]:[PMDETA]:[TX]:[TBE] = 100:5:1:1. The vials underwent nitrogen purging for 10 min under magnetic stirring to eliminate oxygen. Subsequently, the reaction mixture was irradiated with UV light (365 nm and 2 mW cm^–2^) for 6 h at 40 °C. After the reaction, the resulting product was transferred to glass Petri dishes and subjected to a drying process in an oven at 45 °C for 48 h to remove excess monomer and solvent. Prior to characterization, the PLM underwent purification by dissolution in methanol and subsequent dialysis using Spectra/Por dialysis membranes with a molar mass cutoff of 300–500 Da. The dialysis process occurred over 4 days, with the dialysis bath being refreshed every 24 h. The number-average molecular weight (*M̅*_*n*_) of the PLM was determined to be 964 Da based on the analysis of the ^1^H NMR spectrum provided in Figure S2­(b), Supporting Information.

### 2.5 Preparation of GelMA/PLM and GelNB/PLM-Based Hydrogel Films

Stock solutions were prepared, the first containing 10% (w/v) PLM in ethanol and the second containing 2% (w/v) Pluronic F127 in double-distilled water. In a typical test, emulsions were obtained by mixing 0.25 or 0.50 mL of the PLM ethanolic solution with 5 mL of the surfactant solution at 15,000 rpm for 3 min using an IKA UltraTurrax, followed by ultrasound treatment for an additional 15 min [Figure S1­(b), Supporting Information]. The emulsions were then incubated at 37 °C for 1 h, after which 0.5 g of GelMA or GelNB was added to obtain a final gelatin concentration of 10% (w/v). The PLM contents of 5% and 10% (w/w) were selected based on preliminary solubility, processability, and antioxidant screening tests, which showed that concentrations below 5% produced negligible antioxidant enhancement, whereas concentrations above 10% led to phase separation and compromised hydrogel homogeneity. This procedure yielded hydrogels containing 5% (w/w) or 10% (w/w) PLM relative to the gelatin mass, labeled as GelMA/PLM5, GelMA/PLM10, GelNB/PLM5, and GelNB/PLM10. For comparison, PLM-free GelMA and GelNB hydrogels were prepared under identical conditions, totaling six formulations.

Afterward, 4% (mol/mol) of Li-TPO-L (initiator), relative to the number of functionalities previously determined, was added using a 0.8% (w/v) stock solution (the volume ratio of prehydrogel dispersion and initiator solution was approximately equal to 17.5). In addition, when GelNB was considered, 55% (mol/mol) of DTT, relative to the number of NB functionalities, was added as a cross-linker. Finally, the prehydrogel dispersions were injected between two glass plates separated with a silicone spacer (1 mm thick), stored at 4 °C for 30 min to induce physical gelation, and subsequently cross-linked using UV light (365 nm, 10 mW cm^–2^) for 30 min. Figure S1­(b), in the Supporting Information, illustrates the procedure carried out to prepare the hydrogel films.

### 2.6 Hydrogel Characterization

#### 2.6.1 Kinetics of Photo-Cross-Linking for Obtaining GelMA/PLM- and GelNB/PLM-Based Hydrogels

The *in situ* photo-cross-linking rheology test was conducted for all hydrogel formulations using a Physica MCR 350 rheometer (Anton Paar) equipped with plate–plate geometry. In summary, 300 μL of prehydrogel dispersion was loaded onto the equipment, containing Li-TPO-L initiator at 4% (mol/mol) relative to the functionality amount. Subsequently, a strain of 0.1% and an oscillation frequency of 1 Hz were applied, with a gap setting of 0.350 mm. Following a 1 min waiting period, UV light was administered for 10 min utilizing a Novacure 2100 spot curing system (EXFO Photonic Solutions Inc., Hampshire, U.K.), operating at an intensity of 50 mW cm^–2^ and a wavelength range from 320 to 390 nm. The oscillatory measurement was continued for 4 min after deactivating the UV light to observe the postcuring behavior. All measurements were conducted in triplicate at 37 °C.

#### 2.6.2 Swelling Degree and Gel Fraction

Freeze-dried hydrogel discs (8 mm diameter and 1 mm thick) had their mass determined (*m*_1_). The samples were then immersed in phosphate buffer PBS 1× (pH 7.4) and incubated for 24 h at 37 °C, followed by the determination of the mass of the swollen hydrogels (*m*_2_). Excess water was gently removed with a paper towel before m_2_ measurement. The samples were then immersed in ethanol for 24 h, and finally in double distilled water for another 24 h to ensure all additives and non-cross-linked gelatin were removed. After that, the samples were freeze-dried again, and their final mass (*m*_3_) was determined. The degree of swelling (SD, %) and gel fraction (GF, %) were calculated using eqs 1 and 2, respectively. Experiments were conducted in triplicate.

1 $$\mathrm{SD}\;\left(\%\right)=\frac{m_{2}-m_{1}}{m_{1}}\times 100$$

2 $$\mathrm{GF}\;\left(\%\right)=\frac{m_{3}}{m_{1}}\times 100$$

#### 2.6.3 Structural Investigation

The infrared (IR) spectra of GelMA- and GelNB-based hydrogels, along with additives (PLM and F127), were acquired using the FTIR Spectrum Two instrument (PerkinElmer). The instrument was equipped with an Attenuated Total Reflectance (ATR) module and operated at 25 °C. Spectra were obtained by scanning the range of 4000 to 650 cm^–1^, with 16 scans and a resolution of 4 cm^–1^. Furthermore, High Resolution Magic Angle Spinning Nuclear Magnetic Resonance (HR-MAS ^1^H NMR) spectroscopy was performed using a Bruker Avance II 700 MHz spectrometer, which was equipped with a HR-MAS probe featuring ^1^H, ^13^C, and ^119^Sn gradient channels. To conduct this analysis, freeze-dried samples of the cross-linked hydrogels were loaded into a 4 mm MAS rotor with 50 μL of D_2_O and allowed to swell before being spun at 6 kHz. The number of cross-linkable moieties was quantified in terms of the degree of substitution before (DS_1_) and after cross-linking (DS_2_), as detailed in the Supporting Information. Subsequently, the degree of double bond consumption, which reflects the efficiency of cross-linking (DC, %), was determined from the hydrogel spectra using eq 3.

3 $$\mathrm{DC}\left(\%\right)=\left(\frac{{\mathrm{DS}}_{1}-{\mathrm{DS}}_{2}}{{\mathrm{DS}}_{1}}\right)\times 100$$

#### 2.6.4 Molecular Weight between Cross-Links (*M̅*_*c*_) and Cross-Linking Density (ρ_*c*_)

The number-average molecular weight between cross-links (*M̅*_*c*_) was determined using the classical rubber elasticity theory (eq 4), assuming that the network chains in all hydrogels adhere to Gaussian statistic distribution.^45,46^

4 $${\mathrm{M̅}}_{c}=\frac{1}{(\frac{G^{\prime }Q^{1/3}}{\mathrm{cRT}})+\frac{2}{{\mathrm{M̅}}_{n}}}$$

in which *G*′ refers to the storage modulus of the hydrogels (atm), considered approximately as the shear modulus, c is the molar concentration of gelatin (mol L^–1^), R is the universal gas constant (0.082 atm L mol^–1^ K^–1^), *T* is the absolute temperature (K), *M̅*_*n*_ is the number-average molecular weight of the gelatin (g mol^–1^) determined according to Supporting Information, and *Q* is the volumetric swelling ratio (v/v) at the thermodynamic equilibrium (eq 5).

5 $$Q=\frac{\rho_{\mathrm{gel}}\mathrm{SD}}{\rho_{w}}+1$$

in which SD is the mass swelling ratio (w/w), obtained with eq 1; ρ_*w*_ and ρ_gel_ are the densities of the water (1.00 g cm^–3^) and gelatin (1.36 g cm^–3^), respectively. The theoretical density of cross-links (mol cm^–3^) was estimated using eq 6.

6 $$\rho_{c}=\frac{\rho_{\mathrm{gel}}}{{\mathrm{M̅}}_{c}}$$

#### 2.6.5 In-Vitro PLM Release Study and Kinetics

*In-vitro* release studies were carried out using predetermined hydrogel discs (8 mm in diameter and 1 mm thick) containing PLM loads of approximately 1 mg for GelMA/PLM5 and GelNB/PLM5 samples, and 2 mg for GelMA/PLM10 and GelNB/PLM10 samples. These discs were immersed in a release medium comprising 5 mL of PBS 1× (pH = 7.4) at 37 °C. At each specified time interval, 1 mL aliquot was extracted. The absorbance of this aliquot was measured at 256 nm to determine the concentration of PLM in the release medium at that point. Subsequently, the used 1 mL aliquot was discarded, and an equal volume of fresh PBS was replenished into the release medium containing the sample to maintain sink conditions. Hence, the cumulative release of PLM considered the portion of PLM discarded with each withdrawn aliquot, as outlined in eq 7.

7 $$\frac{M_{t}}{M_{\infty }}=\frac{{C_{r}\cdot V}_{r}+\sum_{a=1}^{t}{C_{a}\cdot V}_{a}}{M_{\infty }}$$

In which *C*_*r*_ and *C*_*a*_ are the concentrations (mg mL^–1^) of PLM in the release medium and in the withdrawn aliquot “a”, respectively, at each time; *V*_*r*_ and *V*_*a*_ are the volumes (mL) of the release medium and the aliquot “a”; *M*_*t*_ is the total mass of PLM released from the hydrogel matrix at time “*t*”, and *M*_*∞*_ is the amount of PLM released at thermodynamic equilibrium.

The cumulative release data obtained from the experiments over time (h) underwent empirical mathematical model fitting to gain insights into the release mechanism. First, the Peppas-Sahlin model (eq 8) was employed for analyzing the experimental data owing to its capability to encompass both diffusional and relaxational contributions.^47^

8 $$\frac{M_{t}}{M_{\infty }}=k_{1}\cdot t^{m}+k_{2}\cdot t^{2m}$$

in which *k*_1_ and *k*_2_ are the kinetic constants (h^–m^ and h^–2m^). The term *k*_1_·*t* ^*m*^ denotes the Fickian contribution, while the second term on the right-hand side delineates the Case-II relaxational contribution. The coefficient *m* is the purely Fickian diffusion exponent for a device of any geometrical shape.^47^ Additionally, the Weibull model (eq 9) was used, as it effectively captures the complex release kinetics seen in systems with a burst release followed by a slower, sustained release phase. Its flexibility allows it to describe a wide variety of release mechanisms, including diffusion-controlled release, erosion-controlled release, and their combinations.^48^

9 $$\frac{M_{t}}{M_{\infty }}=\left(1-e^{-\left(t^{\beta }/\alpha \right)}\right)$$

in which α (h^β^) is the scale parameter defining the time scale of the process; and β is the shape parameter, which characterizes the curve as either exponential (β = 1), sigmoidal, with upward curvature followed by a turning point (β > 1), or parabolic, with a higher initial slope and after that consistent with the exponential (β < 1).

Once confirmed that the release profile follows a Fickian diffusion, the experimental data were fitted to Fick’s law to determine the diffusivity coefficient of PLM in the hydrogel matrix. For films where the compound is uniformly dispersed, unsteady-state diffusion in a one-dimensional slap-shaped matrix can be described using Fick’s second law (eq 10).^49^

10 $$\frac{\partial C}{\partial t}=D\frac{\partial^{2}C}{\partial x^{2}}$$

in which *C* is the concentration of the releasing species (here PLM) as a function of *x* and *t*; and *D* is de diffusion coefficient of PLM in the film matrix. The diffusion coefficient *D* was assumed as a constant. Other assumptions include sink condition and a thin planar geometry, where the release through slab edges is neglected. There are different approaches to the analytical solution of eq 10, based on geometric conditions and particularities of the compound in the release system. In this case, the film is in the form of slab-like device, in which eq 11 may be used to provide a reproduction of the release results in the early stage.^50,51^

11 $$\frac{M_{t}}{M_{\infty }}=\frac{4}{l}{\left(\frac{D\cdot t}{\pi }\right)}^{1/2}$$

in which *M*_*t*_ is the amount of additive released at time *t*, *M*_*∞*_ is the amount of additive released at thermodynamic equilibrium and *l* is the thickness of the film. This is an early time approximation that holds for the first 60% of cumulative release, i.e., 0 ≤ *M*_*t*_/*M*_∞_ ≤ 0.6. By linearizing eq 11, and plotting *M*_*t*_/*M*_∞_ *vs t* ^*1/2*^, the diffusivity of PLM, *D*, was easily obtained.

Since PLM release remained consistently below 60% throughout the test period for all samples, the analytical solution of Fick’s second law, as described in eq 11, was used to estimate the diffusion coefficient. This method allows to identify the matrix with the greatest capacity for PLM diffusion. The fitting process for determining the parameters of the empirical models utilized the Levenberg–Marquardt method, implemented through Fityk software, in a manner similar to our previous study.^10^ To assess the goodness of fit between the mathematical models and observed data, the Weighted Sum of Squared Residuals (WSSR) was employed as a key metric. By comparing WSSR values across different models (as detailed in the Supporting Information), we identified the model with the lowest values, ensuring the most accurate representation of release behavior in this study.

#### 2.6.6 Antioxidant Activity via DPPH Radical Scavenging Assay

The capacity of the hydrogel discs to quench the 2,2-diphenyl-1-picrylhydrazyl (DPPH) radical was assessed by measuring the reduction in absorbance of a methanolic 0.15 mM DPPH solution upon contact with the freshly prepared material. Each hydrogel disc (8 mm in diameter and 1 mm thick) was immersed in 2.5 mL of the DPPH solution and incubated in darkness for 2 h. Subsequently, the absorbances of the solutions were recorded at 517 nm using a Shimadzu UV-1900l spectrophotometer. The DPPH radical scavenging activity (%) was determined using eq 13, in which ABS_DPPH_ represents the absorbance of the DPPH solution (blank) and ABS_sample_ denotes the absorbance of the solution containing the hydrogel disc after incubation.

12 $$\mathrm{DPPH}\;\mathrm{scavenging}\;\left(\%\right)=\frac{{\mathrm{ABS}}_{\mathrm{DPPH}}-{\mathrm{ABS}}_{\mathrm{sample}}}{{\mathrm{ABS}}_{\mathrm{DPPH}}}\times 100$$

### 2.7 *In-Vitro* Studies

#### 2.7.1 Cell Lines and Maintenance

Human foreskin fibroblasts (HFF) were cultured in Dulbecco’s Modified Eagle Medium (DMEM) supplemented with 10% (v/v) fetal bovine serum (FBS) and 1% (v/v) antibiotic penicillin/streptomycin and maintained at 37 °C in a 5% CO_2_ atmosphere. The culture medium was refreshed every 2 days, and subculturing was carried out when cells reached 80–90% confluency. For experimentation, hydrogel sheets measuring 8 mm in diameter and 1 mm in thickness were used. Sterilization was achieved through UV–C irradiation (15 mW cm^–2^) for 2 h. Each sample was then incubated in 1 mL of culture medium for 1, 3, and 7 days. The influence of leachable components, primarily PLM, on cell viability and metabolic activity was assessed in triplicate at these time points.

#### 2.7.2 Cell Viability and Morphology

Metabolic activity was determined using the previously reported MTS assay,^52^ which involved the preparation of a mixture containing 16% (v/v) MTS in culture medium, added to the cells. The 96-well plate was incubated in the dark at 37 °C for 2 h with continuous shaking. Absorbance at 490 nm was measured using an EL800 Universal Microplate Reader (BioTek Instruments) with the GEN5 software. A live–dead viability assay was conducted by adding a mixture of 0.2% (v/v) Calcein-AM (Ca-AM) and 0.2% (v/v) propidium iodide (PI) in phosphate-buffered saline (PBS) to the cells. The cells were incubated in the dark for 15 min. Fluorescence microscopy was performed using a confocal microscope Carl Zeiss LSM710 equipped with GFP and Texas Red (TxRed) filters to differentiate between living (green) and dead (red) cells. Image processing for cell viability quantification was carried out using Fiji software.

### 2.8 *In-vivo* Studies

This phase of the study was approved by the Research Ethics Committee of “Grigore T. Popa” University of Medicine and Pharmacy in Iaşi and followed ethical guidelines for laboratory animal research (Law no. 206/27 May 2004, EU/2010/63–CE86/609/EEC). The study was conducted at the Advanced Research and Development Center for Experimental Medicine (CEMEX), “Grigore T. Popa” University of Medicine and Pharmacy, Iaşi (certificate no. 2/22.09.2017).

#### 2.8.1 Animal Model and Maintenance

The *in vivo* experiment was conducted using adult male Wistar rats (6–8 weeks old, weighing 350–400 g). The animals were housed separately in polypropylene cages for 7 days under controlled conditions, including a constant temperature (23 ± 2 °C), relative humidity (37–60%), and a 12 h light/dark cycle. They were fed standard pellets and had ad libitum access to water. The rats were divided into four groups and observed for 18 days following surgical excision. The most promising PLM-loaded gelatin-based hydrogel was selected based on its bioactive and physicochemical properties, as well as the concentration of PLM that did not exhibit cytotoxicity. A sterile gauze negative control (Cotton gauze) and a commercial sodium alginate-based positive control (Sorbalgon) were also included. A click dressing was used to protect the treated surface, as previously described.^53^

#### 2.8.2 Qualitative and Quantitative Analysis of the *In Vivo* Wound Healing Process

The protocol followed a standard procedure: a 2 cm diameter wound was created and excised, and dressings were replaced every 3 days. Macroscopic examination of the wounds was performed, including imaging and measurement of wound area using a developed code in Python. The wound healing rate was correlated with the contraction rate (CR, %), calculated by eq 13.

13 $$\mathrm{CR}\;\left(\%\right)=\left(\frac{A_{0}-A_{t}}{A_{0}}\right)\times 100$$

in which “*A*” is the wound area, with “0” representing the initial measurement on day 0, and “*t*” representing measurements taken on subsequent days (3, 6, 9, 12, 15, or 18). Biopsy samples were collected on days 6, 12, and 18 for histopathological analysis.

The wound contraction rate provides data on the reduction in wound area over time, with a higher contraction rate indicating improved healing efficiency. At the end of the experiment, the animals were euthanized in a humane manner to avoid any suffering. The protocol ensured that the animals experienced no discomfort, with swift induction of unconsciousness, cessation of cardiac function, loss of respiration, and eventual death. The rats were food-deprived and then anesthetized with isoflurane 24 h before euthanasia. After the final biopsies were collected, the rats were euthanized through intracardiac injection of 1–2 cc of potassium chloride (KCl) while under isoflurane anesthesia. This method, combined with isoflurane anesthesia, ensures a painless procedure for the animals.

#### 2.8.3 Histological Analysis

Skin samples, including the wound area and surrounding margins, were collected and fixed in 10% buffered formaldehyde for 48 h. Histological processing was performed using the paraffin-embedding method with a Leica TP1020 tissue processor (Leica Microsystems GmbH, Germany). Tissue sections were cut at a thickness of 5 μm using a SLEE CUT 6062 microtome (SLEE Medical GmbH, Germany), followed by deparaffinization and staining with Masson’s trichrome (hematoxylin-eosin-methylene blue). Histological analysis was conducted using a Leica DM750 optical microscope equipped with a Leica ICC50 HD digital camera (Leica Microsystems GmbH, Germany). Micrographs were captured with the Leica Application Suite (LAS) software, version 4.2. All procedures for histological processing, imaging, and interpretation were performed under standardized conditions across all experimental groups.

### 2.9 Statistical Analysis

Quantitative measurements were conducted in triplicate and presented as mean values ± standard deviation. To assess the statistical significance of differences among group averages, a one-way analysis of variance (ANOVA) was performed. Tukey’s test was subsequently employed to identify statistically significant discrepancies between samples (*p* < 0.05), denoted by distinct letters accompanying each mean value.

## 3 Results and Discussion

### 3.1 Photo-Cross-Linking Kinetics of GelMA/PLM and GelNB/PLM

Figure 2(a) illustrates the time-dependent evolution of the storage modulus (*G*′) for GelMA and GelNB hydrogel forming solutions under UV irradiation, starting 1 min into the experiment. Initially comparing GelMA and GelNB without PLM, it is evident that GelNB exhibits a notably more rapid increase in *G*′ compared to its counterpart. Within the initial seconds of UV exposure, GelNB reaches a *G*′ plateau of approximately 3 kPa, whereas GelMA demonstrates gradual growth, ultimately reaching a final *G*′ of 5 kPa after UV exposure. It was anticipated that GelMA hydrogels would demonstrate higher stiffness (*G*′) owing to the chain-growth polymerization mechanism, which facilitates the linking of various functionalities within the same junction knot.^54^ In contrast, for thiol–ene systems (GelNB), which adhere to a step-growth polymerization mechanism, the situation differs. Here, the number of functionalities linked within one junction knot hinges on both the quantity and spatial arrangement of thiol groups on the cross-linker molecule, dictated by the reaction of complementary functionalities.^37,43,55^ Moreover, in the case of GelMA hydrogels, several methacryloyl moieties on the same macromolecular chain can react with each other, resulting in primary cycles and associated network imperfections.^56^ This effect is less pronounced for GelNB due to the orthogonal cross-linking occurring between complementary functionalities. These reactional differences are schematized in Figure 4(a,b).

**Figure 2:**
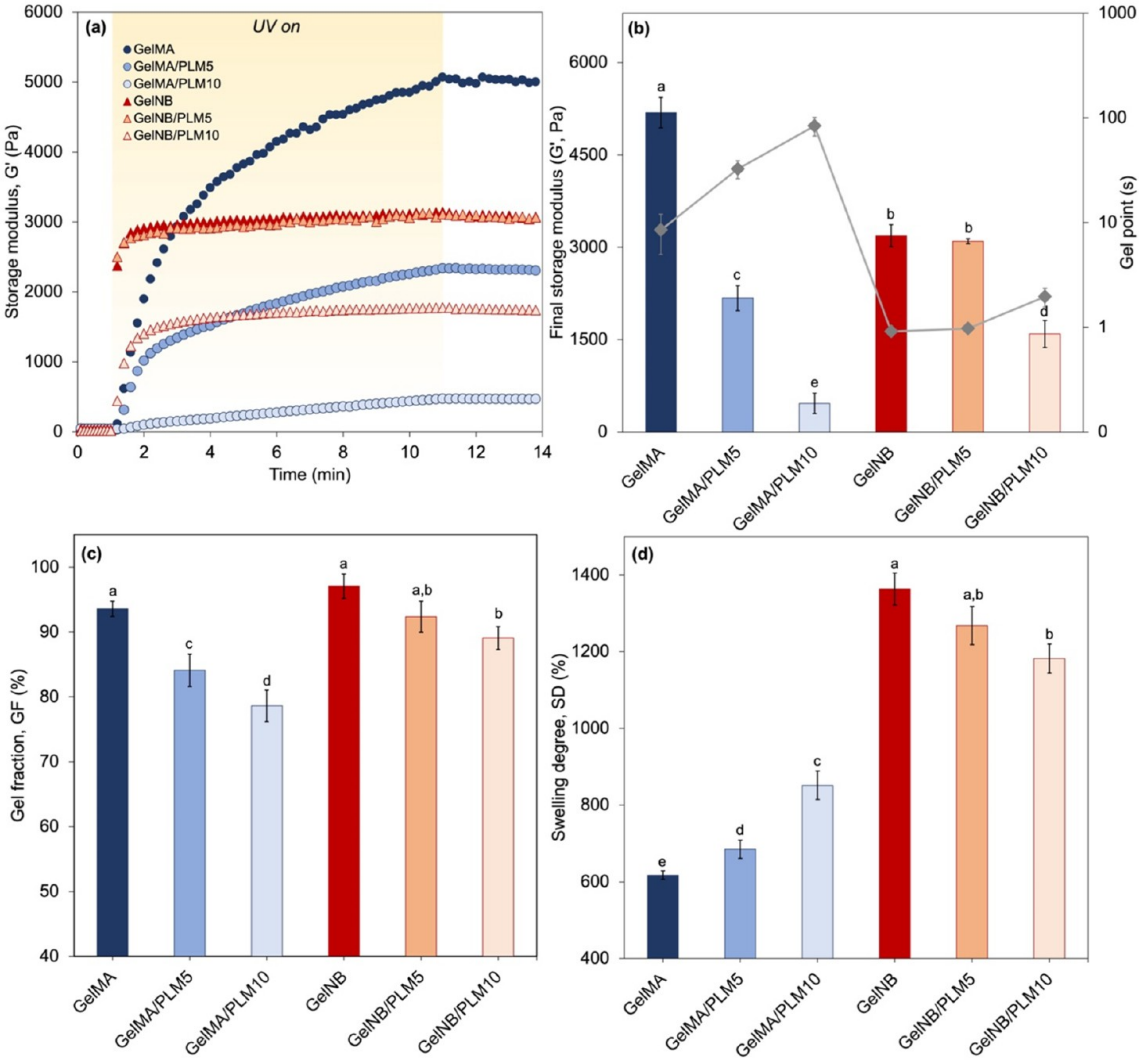
(a) Storage modulus (*G*′, Pa) plotted against exposure time to UV light (320–390 nm, 50 mW cm^–2^) of hydrogel-forming solution containing 4 mol % of Li-TPO-L as the initiator; (b) final storage modulus (*G*′, Pa) after the *in situ* cross-linking rheometric experiment, showcased through bar graphs, with the gel point (s) determined as the crossover between storage and loss moduli after UV light exposure, represented by the gray dotted line; (c) gel fraction (GF, %); and (d) swelling degree (SD, %) measured after 24 h of incubation in PBS (pH 7.4) at 37 °C. Different letters (a, b, c, d, etc.) indicate statistically different groups according to one-way ANOVA followed by Tukey’s test (*p* < 0.05).

Figure 2(b) delineates the final *G*′ values alongside the precise times at which the crossover between *G*′ and *G*″ occurred, representing the gel point of the hydrogels. GelNB achieves gelation in less than 1 s, whereas GelMA necessitates approximately 10 s of UV exposure to achieve its gel point. These variances were also anticipated due to the disparate photo-cross-linking chemistries inherent to each functional group. Since GelMA undergoes cross-linking through a chain-growth polymerization mechanism, involving successive radical additions, an induction phase upon UV irradiation due to oxygen inhibition is observed. Before polymerization initiates, radicals must consume oxygen, unlike in step-growth thiol–ene systems (GelNB), leading to the observed induction phase in the cross-linking reaction.^57^ These differences between both chemistries are further emphasized when PLM is incorporated in the hydrogel forming solution before cross-linking, in which the incorporation of PLM had a significant influence on *G*′ and gel point.

Particularly, this effect was more pronounced for GelMA-based hydrogels. Figure 2(a) illustrates a notable alteration in the *G*′ profiles over time following the introduction of varying proportions of PLM. Specifically, there was a significant decrease (*p* < 0.05) in *G*′ from 5 kPa (GelMA) to approximately 2 and 0.5 kPa for GelMA/PLM5 and GelMA/PLM10, respectively. Figure 2(b) further underscores a substantial increase (*p* < 0.05) in gel point, extending from approximately 10 s (GelMA) to roughly 100 s with the highest PLM proportion. These findings suggest a potent inhibitory effect on polymerization induced by PLM. With allylic hydrogens scattered throughout its structure,^44^ PLM readily lends itself to radical abstraction, thus increasing the induction period for GelMA photo-cross-linking [illustrated in Figure 4(a)]. Consequently, as PLM tends to impede radical propagation, hindering the linking of various functionalities within the same junction knot, the final *G*′ experienced a pronounced reduction. This phenomenon was not observed in the comparison between GelNB and GelNB/PLM5. In this scenario, *G*′ and the gel point were statistically equivalent (*p* > 0.05), underscoring the robustness of the thiol–ene step-growth polymerization mechanism. However, it is noteworthy that with the incorporation of 10% PLM, there was a significant reduction in *G*′ and an increase in the gel point (*p* < 0.05). Despite this, GelNB/PLM10 exhibited a stiffness roughly three times higher than GelMA/PLM10 (*p* < 0.05).

### 3.2 Gel Fraction, Swelling Degree, and Morphology of the GelMA/PLM- and GelNB/PLM-Based Hydrogels

The distinct chain growth mechanisms involved in the cross-linking process also exerted significant influence on fundamental physical properties of hydrogels, as evidenced by the gel fraction (GF) [Figure 2(c)] and equilibrium mass swelling degree (SD) [Figure 2(d)]. While the GF of GelMA and GelNB were statistically similar (*p* > 0.05), both exceeding 90% and indicating efficient cross-linking, the incorporation of PLM led to a progressive reduction in GF, with the magnitude of this effect depending on both the gelatin functionalization and PLM content. While GF remained relatively stable at around 90% for all GelNB samples, it decreased to approximately 80% for GelMA/PLM10, underscoring the hypothesis that PLM indeed significantly inhibited the chain growth polymerization in GelMA hydrogels, thereby impeding effective cross-linking of gelatin chains. In GelNB-based hydrogels, a modest decreasing trend in GF was observed from GelNB to GelNB/PLM5 and GelNB/PLM10, although all values remained close to 90%, indicating that thiol–ene cross-linking is less sensitive to PLM-induced inhibition than the methacrylate chain-growth system. This behavior contrasts with the rheological response, in which GelNB and GelNB/PLM5 display statistically similar *G*′ values, suggesting that small variations in GF do not necessarily result in large changes in macroscopic stiffness for these networks. Importantly, the direct comparison between GelMA/PLM5 and GelNB/PLM5 confirmed a statistically significant difference between the two systems (*p* < 0.05), highlighting that even at the same PLM content, the underlying cross-linking chemistry leads to distinct structural outcomes.

Concurrently, the SD, assessed for the various GelMA- and GelNB-based hydrogels in PBS at 37 °C [Figure 2(d)], revealed intriguing behavior. Initially, a substantial disparity was observed between hydrogel samples without PLM. GelMA exhibited an SD value of around 600%, whereas GelNB displayed an SD exceeding double that value (>1300%), highlighting its significant water absorption capacity, particularly suitable for applications such as wound dressings that necessitate high exudate absorption.^4^ These contrasting behaviors may be attributed to the less densely cross-linked network formed in GelNB, enabling greater water absorption, a direct consequence of the step-growth polymerization mechanism of GelNB, resulting in a more homogeneous network compared to the heterogeneous network generated in GelMA [Figure 4(a) and (b)]. During the chain-growth polymerization of GelMA, the formation of hydrophobic oligomethacryloyl and irregular chain growth lead to the establishment of a densely cross-linked network, thereby resulting in lower water uptake. This trend aligns with observations from prior studies involving GelNB/SH versus GelMA scaffolds produced through extrusion-based 3D printing.^58^ Furthermore, similar patterns were noted in comparisons between allylated gelatin cross-linked with DTT and GelMA hydrogels,^54^ as well as between recombinant collagen type I functionalized with NB/SH and its methacryloyl-functionalized counterpart.^59^

Another pertinent aspect regarding SD involves the impact of PLM on both gelatin functionalization approaches. Increasing the proportion of PLM resulted in a statistically significant rise (*p* < 0.05) in SD for GelMA, escalating from approximately 600% to around 700% and 850% for GelMA/PLM5 and GelMA/PLM10, respectively. The inhibitory effect of PLM on radical propagation significantly elevated SD due to the reduction in cross-linking density, a point that will be further discussed. It is well established that lower cross-linking densities tend to correlate with higher SD and lower *G*′ values, hence this behavior was anticipated. Conversely, GelNB and GelNB/PLM5 did not exhibit statistically significant differences (*p* > 0.05) in SD, despite a clear downward trend in SD with PLM incorporation, culminating in a statistically significant reduction (*p* < 0.05) when comparing GelNB and GelNB/PLM10. This opposite behavior between the two systems can be explained by the distinct roles played by PLM in each network. In GelMA, PLM predominantly acts as a radical scavenger, directly inhibiting chain-growth cross-linking and reducing GF. The resulting decrease in network connectivity increases free volume and mesh size, thereby enhancing water uptake and leading to higher SD. In contrast, in GelNB, where thiol–ene step-growth cross-linking is less affected by PLM, the hydrophobic nature of the oligomer becomes the dominant factor. The incorporation of PLM reduces the effective hydrophilicity of the matrix, decreasing the affinity of the network for water molecules. As a result, even with a slight decrease in GF, the SD of GelNB-based hydrogels diminishes with increasing PLM content. Nonetheless, all GelNB samples still exhibited SD values surpassing 1100%, which represents an impressive range for the intended application.

The distinct morphological features observed through SEM further corroborate the influence of the underlying photo-cross-linking mechanisms and the presence of PLM on the physical properties of the hydrogels. As depicted in Figure 3(a), GelMA-based hydrogels exhibited a porous cross-sectional structure. Conversely, GelNB-based hydrogels displayed a markedly different morphological profile, as shown in Figure 3(b). Overall, the surfaces appeared smoother, and the cross-section exhibited a dense, compact structure with limited porosity, an outcome consistent with the step-growth thiol–ene polymerization mechanism, which yields more homogeneous and uniformly cross-linked networks.^60^ Interestingly, the addition of 10% PLM induced the appearance of phase-separated domains and voids, particularly at the surface, although without substantially altering the overall network homogeneity dictated by the thiol–ene chemistry. It is important to emphasize that, in this present study, the hydrogels were used as topical dressings and replaced every 3 days, their function relied primarily on sustaining antioxidant release and maintaining a protective barrier rather than supporting cell infiltration or long-term tissue ingrowth. Thus, although GelNB exhibits limited porosity, this morphology is fully compatible with the intended therapeutic role of the material.

**Figure 3:**
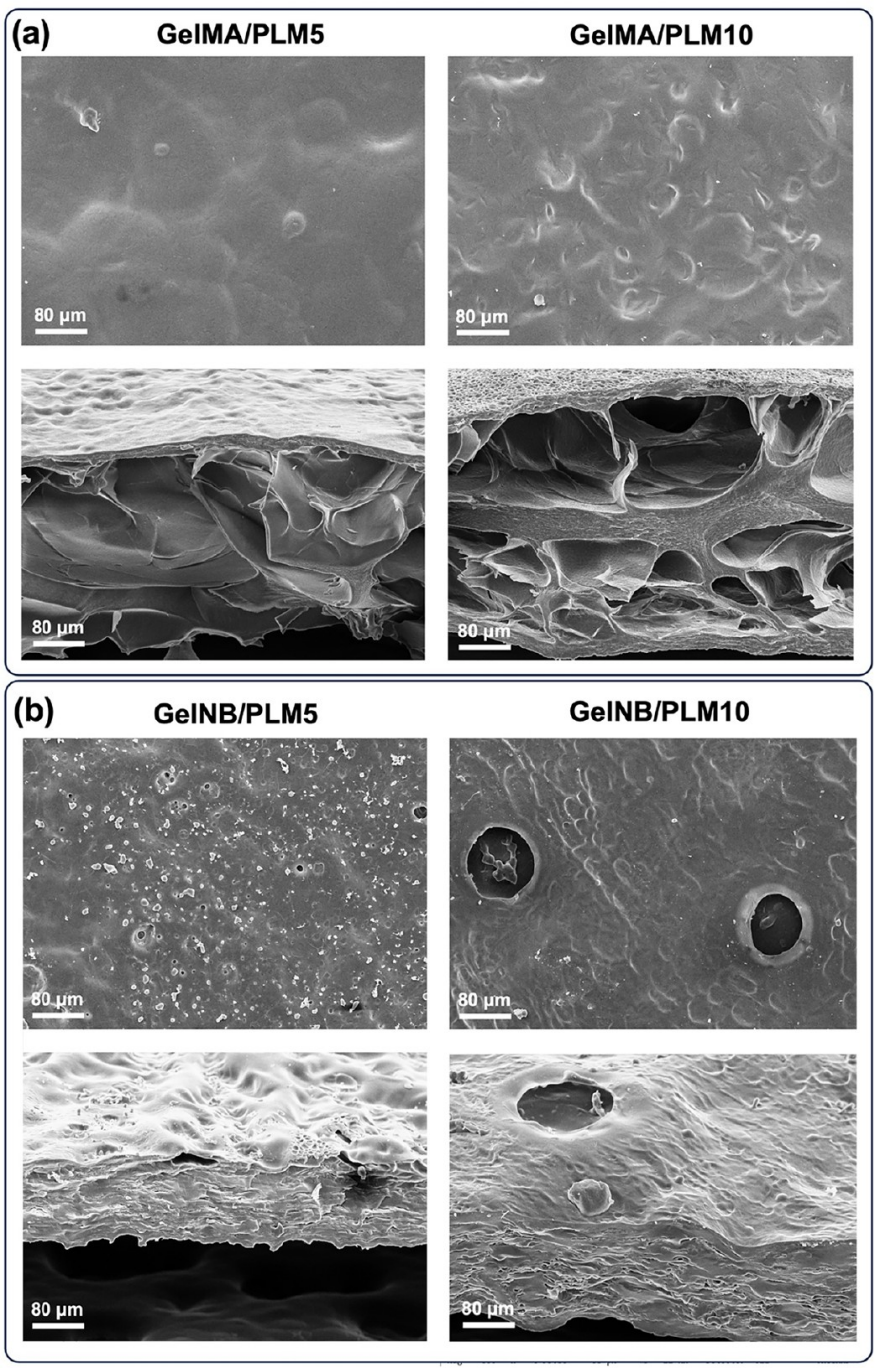
SEM micrographs of (a) GelMA/PLM and (b) GelNB/PLM hydrogels showing surface and cross-sectional morphology at different PLM concentrations.

### 3.3 Hydrogels’ Structure and Cross-Linking Efficiency

Figure 4(a) illustrates the chain-growth polymerization mechanism during the cross-linking of GelMA. The incorporation of PLM is expected to inhibit radical propagation, as its allylic sites efficiently scavenge free radicals, thereby reducing the extent of chain growth and contributing to the lower cross-linking density observed experimentally. In contrast, Figure 4(b) represents the orthogonal and more uniform network generated by thiol–ene step-growth photopolymerization. In this mechanism, cross-linking proceeds through stoichiometric reactions between complementary thiol and norbornene groups, producing a more homogeneous architecture and significantly fewer primary loops. Because thiol–ene reactions rely on much lower steady-state radical concentrations than chain-growth systems, the influence of PLM as a radical scavenger is substantially attenuated in GelNB formulations. This mechanistic distinction explains why the addition of PLM has a pronounced effect on GelMA gels but only a moderate impact on GelNB networks, except at the highest PLM loading.

**Figure 4:**
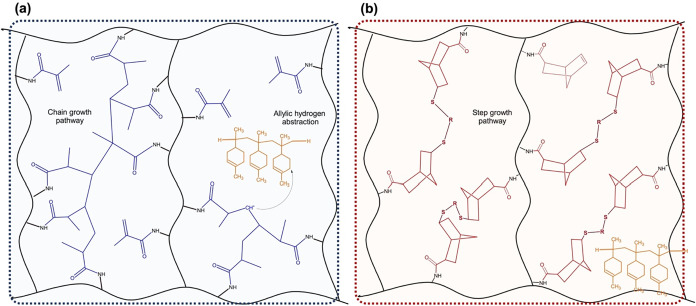
Schematic representation of the (a) chain-growth (GelMA) and (b) step-growth (GelNB) photo-cross-linking pathways and the influence of PLM on each system.

As discussed earlier, these differences in reaction mechanisms result in three-dimensional networks with distinct physicochemical properties. To support these findings, number-average molecular weight between cross-links (*M̅*_*c*_) and cross-link densities (ρ_*c*_) were determined using the rubber-elasticity theory, with the values reported in Figure 5(a). GelMA-based hydrogels have been previously reported to have *M̅*_*c*_ values ranging between 2.8 and 5.3 kg mol^–1^ and ρ_*c*_ between 2.55 × 10^–4^ and 4.70 × 10^–4^ mol cm^–3^,^61^ which align closely with those obtained in this study, despite differences in preparation methods. Incorporating PLM into the GelMA matrix led to a significant increase in *M̅*_*c*_ and a notable reduction in ρ_*c*_, irrespective of the proportion of PLM used. For instance, the ρ_*c*_ decreased (*p* < 0.05) from 3.07 × 10^–4^ (GelMA) to approximately 6.6 × 10^–5^ mol cm^–3^ (GelMA/PLM10), underscoring the significant impact of PLM on radical propagation inhibition and consequently reducing cross-linking density. In contrast, comparing GelNB and GelNB/PLM5 revealed no significant changes (*p* > 0.05) in *M̅*_*c*_ and ρ_*c*_, consistent with previous findings on physicochemical properties.

**Figure 5:**
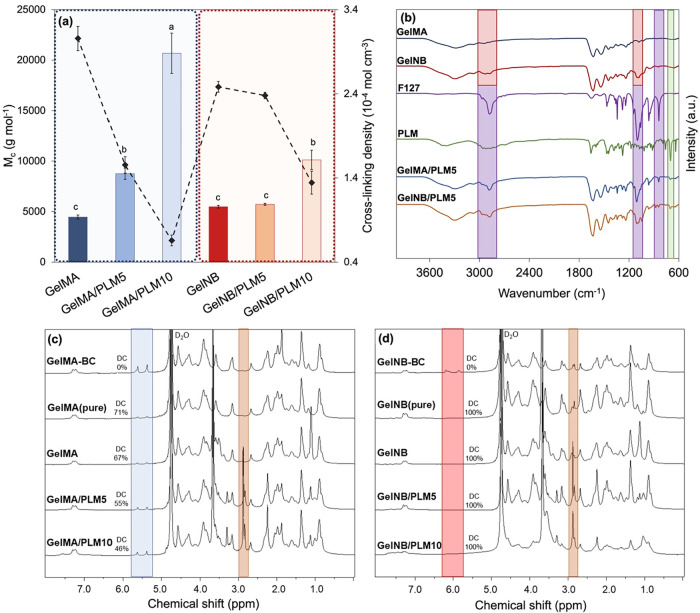
(a) determination of the number-average molecular weight between cross-links by the bar chart, while the estimated cross-linking density is represented by the dashed line; (b) Fourier transform infrared spectra (FT-IR) for the functionalized gelatins and their comparison with GelMA/PLM5 and GelNB/PLM5 samples; and HR-MAS ^1^H NMR spectra of all (c) GelMA- and (d) GelNB-based hydrogel samples, along with the functionalized gelatin before cross-linking (referred to as GelMA-BC or GelNB-BC) and hydrogels without any additive referred to as “pure” in parentheses. Different letters (a, b, c, d, etc.) indicate statistically different groups according to one-way ANOVA followed by Tukey’s test (*p* < 0.05).

In Figure 5(b), three colored regions highlight characteristic bands of specific functional groups in both GelMA and GelNB. The pink region (around 2900 cm^–1^) corresponds to C–H stretching, characteristic of alkyl and alkene groups, which may be associated with the introduced functional groups. Compared with GelMA spectra, a clear distinction is observed in the shape and intensity of the GelNB signals, where signals around 2850–2950 cm^–1^ are attributed to the C–H stretching vibrations of methylene (−CH_2_) and methyl (−CH_3_) groups of its bicyclic ring,^62^ which manifest prominently in this spectral range. The purple-marked region, around 2800 cm^–1^, corresponds to C–H (sp^3^) stretching related to the alkyl chains of the PPO block,^63^ which is a block of F127. The presence of ether bonds in the polymer structure is confirmed by C–O–C stretching, with an intense band around 1050–1150 cm^–1^,^63^ characteristic of the ethylene oxide and propylene oxide units. These signals are also observed in the spectra of GelMA/PLM5 and GelNB/PLM5, where F127 is added as an emulsifier for PLM. Finally, the green-highlighted region (around 890 cm^–1^) corresponds to vibrations associated with the endocyclic unsaturation present in the PLM molecule.^64^ This signal is also observed, albeit with lower intensity, in the spectra of GelMA/PLM5 and GelNB/PLM5. Thus, the presence of the incorporated additives in the dried hydrogels is confirmed.

Figure 5(c,d) show the HR-MAS ^1^H NMR spectra of the entire array of prepared hydrogels, encompassing samples without an additive [GelMA­(pure) or GelNB­(pure)], as well as samples of functionalized biopolymers lacking any cross-linking [GelMA-BC and GelNB-BC]. These spectra facilitate a meticulous comparison of the extent of double bond consumption (DC) from methacryloyl and norbornene functionalities, prominently highlighted between 5 and 6.5 ppm, as elucidated in the detailed macromers’ structural analysis in the Supporting Information. The DC for GelMA­(pure) [Figure 5(c)], in milli-Q water, without additional additive, was determined as 71%, indicative of a certain challenge in achieving heightened levels of cross-linking efficiency. Comparatively, Pamplona et al. have reported DC of GelMA in PBS as 83.6%, and in DMEM media supplemented with 1 g L^–1^ and 4.5 g L^–1^ of glucose as 73.3% and 69.1%, respectively,^65^ while Billiet et al. reported a DC of around 60% for GelMA (DS ∼ 66%) cross-linked in PBS in similar conditions to this study.^66^

The cross-linking efficiency in the presence of solely F127 surfactant (GelMA) experienced a marginal decline from 71% to 67%, suggesting minimal impact due to its low proportion. However, upon the inclusion of PLM, the DC decreased to 55% and 46% for GelMA/PLM5 and GelMA/PLM10, respectively, consistent with the preceding discussion regarding propagation inhibition stemming from allylic hydrogen abstraction from its backbone, as schematized in Figure 4(a). In contrast, Figure 5(d) highlights the complete disappearance of unsaturation signs of norbornene groups (between 6.0 and 6.4 ppm) post-cross-linking, indicating an efficient process regardless of the additive type and proportion. Although a DC of 98% for GelNB/PLM10 remains notably high, the results of previously discussed physicochemical properties suggest a negative effect from the inclusion of 10% PLM into the GelNB. Consequently, the DC findings suggest that the mere presence of PLM in larger proportions can modify the material’s properties even without substantially impacting cross-linking. This further implies that the step-growth thiol–ene photopolymerization depicted in Figure 4(b) proceeds without interference from PLM.

### 3.4 PLM Release Behavior from Hydrogel Films

Figure 6(a) provides the release profiles of PLM in PBS medium (pH = 7.4) at 37 °C from both gelatin-based hydrogel matrices with different PLM concentrations. Experimental results are represented by dots, while solid lines indicate the Weibull model, selected for its optimal fit, as detailed in the Supporting Information. Comparing GelMA- and GelNB-based hydrogels over the ∼18 h test period, clearly PLM exhibited easier release from GelNB structures. This phenomenon can be attributed to GelNB’s cross-linking mechanism via the step-growth thiol–ene route, ensuring a more uniformly cross-linked matrix [Figure 4(b)]. As previously discussed, GelNB hydrogels demonstrated nearly double the swelling capacity of GelMA hydrogels, facilitating the release of the bioactive agent during the swelling phase.

**Figure 6:**
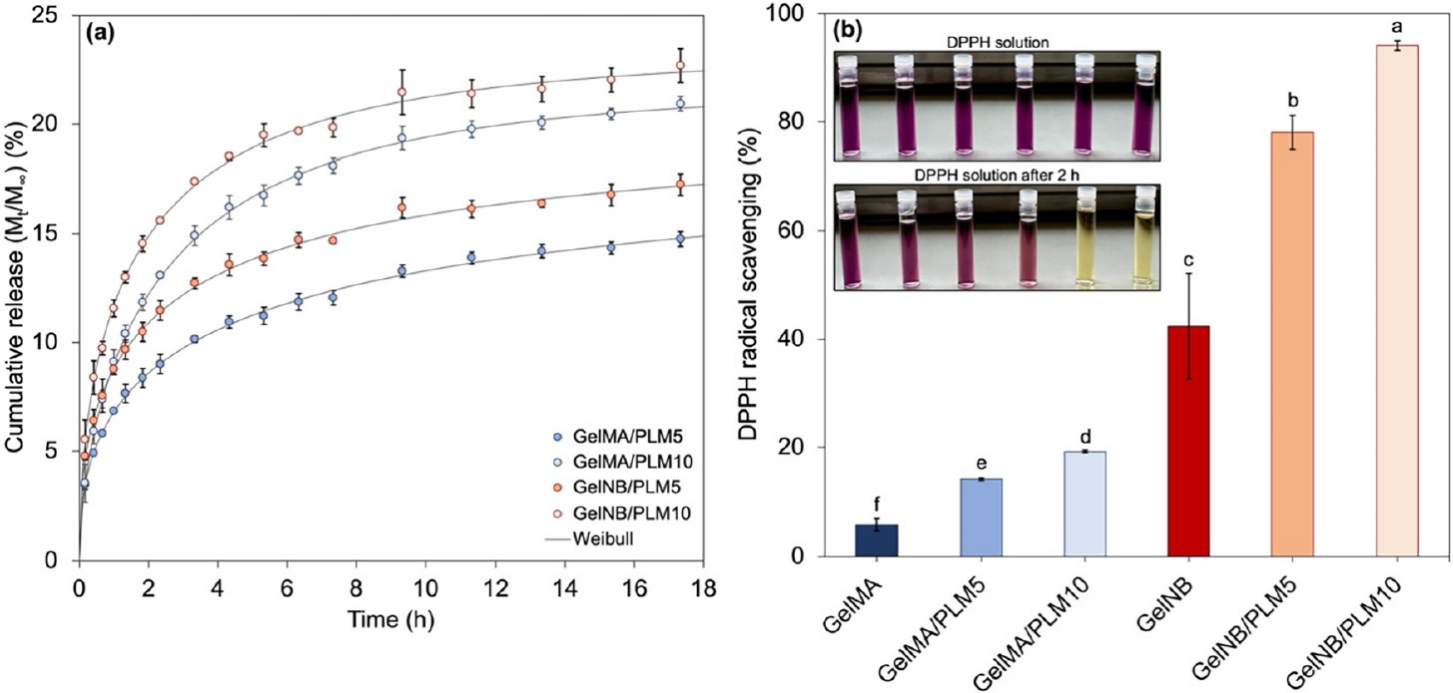
(a) PLM release profiles from GelMA- and GelNB-based hydrogel discs; (b) pictures and percentage of DPPH radical scavenging after incubating the solutions containing hydrogel discs for 2 h in a dark environment. Different letters (a, b, c, d, etc.) indicate statistically different groups according to one-way ANOVA followed by Tukey’s test (*p* < 0.05).

Conversely, the nonregular and hydrophobic oligomethacryloyl chains formed through GelMA’s photo-cross-linking may trap PLM more effectively, delaying its release. In addition, the inherently hydrophobic and oligomeric nature of PLM promotes strong partitioning within the gelatin network, reducing its diffusivity and contributing to the relatively low cumulative release (<25%). From the fitted Weibull model, it was estimated that, at thermodynamic equilibrium, the maximum cumulative release of hydrogels would be approximately 18% and 21.5% for GelMA/PLM5 and GelMA/PLM10, respectively; and 19.3% and 23.2% for GelNB/PLM5 and GelNB/PLM10, respectively. These values were predicted using Weibull model to be reached after 9 days. Despite this limited release, PLM retained within the hydrogel matrix may still exert localized antioxidant effects at the wound interface, scavenging reactive oxygen species (ROS) upon direct contact with tissues, which is consistent with the enhanced *in vivo* wound closure observed for GelNB/PLM5 (Figure 9).

Given that empirical models often encompass multiple mechanisms, the shape parameter of the Weibull model, β, was employed to delve deeper into the release profile and gain insights into the release mechanism of GelMA- and GelNB-based hydrogels. GelMA/PLM5 and GelMA/PLM10 exhibited β values of 0.449 and 0.627, respectively, while GelNB5 and GelNB10 presented β values of 0.450 and 0.542, respectively. Since all β values were below 1, the model confirmed an initial accelerated release characteristic of systems undergoing burst release followed by sustained release phases.^48^ Indeed, experimental profiles indicated a burst during the initial 2 h of the experiment, with a high first-order derivative, followed by a gradual reduction in this rate of variation in subsequent hours, signifying a more sustained release of PLM from hydrogels. This profile is particularly promising for the immediate release of antioxidant agents during acute phases of wound inflammation,^67^ followed by a gradual release, minimizing the need for dressing changes and thereby enhancing patients’ quality of life.

Furthermore, all β values lower than 0.75 suggest Fickian diffusion mechanism,^48,68^ a concentration-dependent phenomenon elucidating the higher cumulative release with increased PLM load in the same gelatin matrix. Therefore, in hydrogels where the compound is uniformly dispersed throughout the matrix, unsteady-state drug diffusion in a one-dimensional slab-shaped matrix can be described using Fick’s second law (eq 10).^69^ The analytical solution of Fick’s second law (eq 11) was employed to determine de diffusivity of PLM in the hydrogel matrices, *D*, as 1.94 × 10^–9^ cm^2^ s^–1^ and 4.17 × 10^–9^ cm^2^ s^–1^ for GelMA/PLM5 and GeMA/PLM10, respectively; and 2.78 × 10^–9^ cm^2^ s^–1^ and 5.83 × 10^–9^ cm^2^ s^–1^ for GelNB/PLM5 and GelNB/PLM10, respectively. Such differences were anticipated, as the varying proportions of PLM and different gelatin functionalization influenced the photo-cross-linking differently, and therefore the final hydrogel’s structure–property relationship. In comparison, the *D* values of proteins from recombinant GelMA-based hydrogels were considerably higher, ranging from 5 × 10^–7^ to 4 × 10^–8^ cm^2^ s^–1^, due to the affinity of the proteins with the release medium.^70^

### 3.5 Antioxidant Activity

The DPPH radical scavenging capabilities of hydrogels are depicted in Figure 6(b). Both GelMA and GelNB control hydrogels exhibit antioxidant properties, albeit to varying extents. Within the 2 h of exposure, GelMA demonstrates a modest DPPH radical scavenging capacity of only 6%, whereas GelNB exhibits a considerably higher efficacy, scavenging approximately 42% of DPPH radicals. This notable disparity in antioxidant activity between GelMA and GelNB hydrogels suggests distinct mechanisms at play. The heightened antioxidant activity observed in GelNB hydrogels could be attributed to the presence of DTT as a cross-linking agent. DTT is known to reduce disulfide bonds to free thiols,^71^ and to chelate divalent cations.^72^ Moreover, DTT has been demonstrated to be a powerful protective agent against oxidative damage in either zygotes or blastocysts.^73^ The formation of thioether bonds with hydroxyl groups near sulfur atoms during photo-cross-linking further contributes to the retained antioxidant potential. These hydroxyl groups can participate in antioxidant reactions by donating hydrogen atoms to free radicals,^28^ thereby neutralizing their reactivity. Although not directly measured in this study, such antioxidant activity may influence classical oxidative stress pathways, including modulation of ROS levels and activation of endogenous defense mechanisms such as Nrf2/HO-1, SOD, and CAT, which play key roles in redox homeostasis during tissue repair.^74,75^

The addition of PLM led to a significant increase (*p* < 0.05) in the DPPH radical scavenging activity of GelMA, elevating it from 6% to approximately 14% and 19% for GelMA/PLM5 and GelMA/PLM10, respectively. This concentration-dependent enhancement underscores the role of PLM as an antioxidant additive in hydrogels. GelNB exhibited a remarkable rise (*p* < 0.05) in antioxidant activity from 42% (GelNB) to approximately 80% and 90% for GelNB/PLM5 and GelNB/PLM10, respectively. The color change observed in the DPPH radical solutions before and after 2 h contact with GelNB samples containing PLM further supports these findings. These results align with previous discussions regarding the inhibition of the chain-growth pathway during the photo-cross-linking of GelMA incorporating PLM. The oligomer effectively hampers this process by donating allylic hydrogens from its structure to impede photo-cross-linking propagation [Figure 4(a)]. Consequently, when GelMA hydrogels containing PLM were evaluated for their ability to inhibit new radicals, such as DPPH, their activity was diminished due to the prior scavenging action of PLM during hydrogel preparation. Conversely, PLM does not significantly impede the photo-cross-linking process via step-growth pathway. Thus, its antioxidant activity remains intact during hydrogel preparation, resulting in the exceptional radical scavenging efficacy of PLM-incorporated GelNB hydrogels.

### 3.6 *In-Vitro* Cytotoxicity

While the antioxidant activity of the hydrogels increased with higher PLM content, Figure 7 highlights the importance of carefully selecting the additive concentration, given its potential influence on cellular responses. Both the control hydrogels (GelMA and GelNB) and those enriched with 5% (w/w) PLM maintained cells in a healthy morphology, characterized by elongated shapes and high density, as evidenced by the predominance of green fluorescence over 1, 3, and 7 days. Quantitative analysis presented in Figure 8(a) further reinforces these observations, showing the percentage of cell viability obtained through live/dead image processing using Fiji software. Both the control hydrogels and those supplemented with 5% (w/w) PLM sustained cell viability above 85% throughout the 7-day testing period, a level deemed cytocompatible according to ISO 10993–1 standards,^76^ which consider viability above 70% acceptable. This finding is corroborated by the analysis of metabolic activity depicted in Figure 8(b), which exhibits a similar trend. Interestingly, a slight increase in viability was observed for GelNB/PLM5 compared to GelNB. This can be explained by two synergistic effects: (i) PLM slightly reorganizes the network, reducing early exposure to residual DTT at the hydrogel surface, and (ii) its intrinsic antioxidant activity mitigates oxidative stress at the cell-material interface, collectively leading to a modest improvement in metabolic activity. GelMA (control) exhibited higher cell viability and metabolic activity compared to their GelNB counterpart. This discrepancy may be attributed to the presence of residual DTT in GelNB, which has been shown to slightly reduce cell viability depending on its concentration and duration of contact with cells.^77,78^ Notably, this effect did not translate into cytotoxicity, as all GelNB-based samples, including those with 5% PLM, maintained viability and metabolic activity well above ISO-compliant levels.

**Figure 7:**
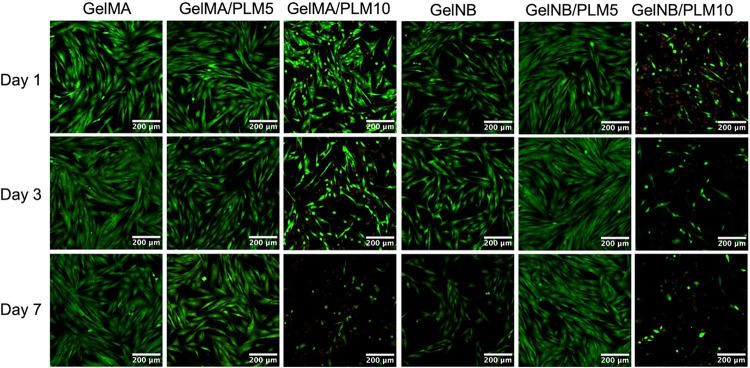
Qualitative assessment of cell viability via a live/dead assay after 1, 3, and 7 days of incubation.

**Figure 8:**
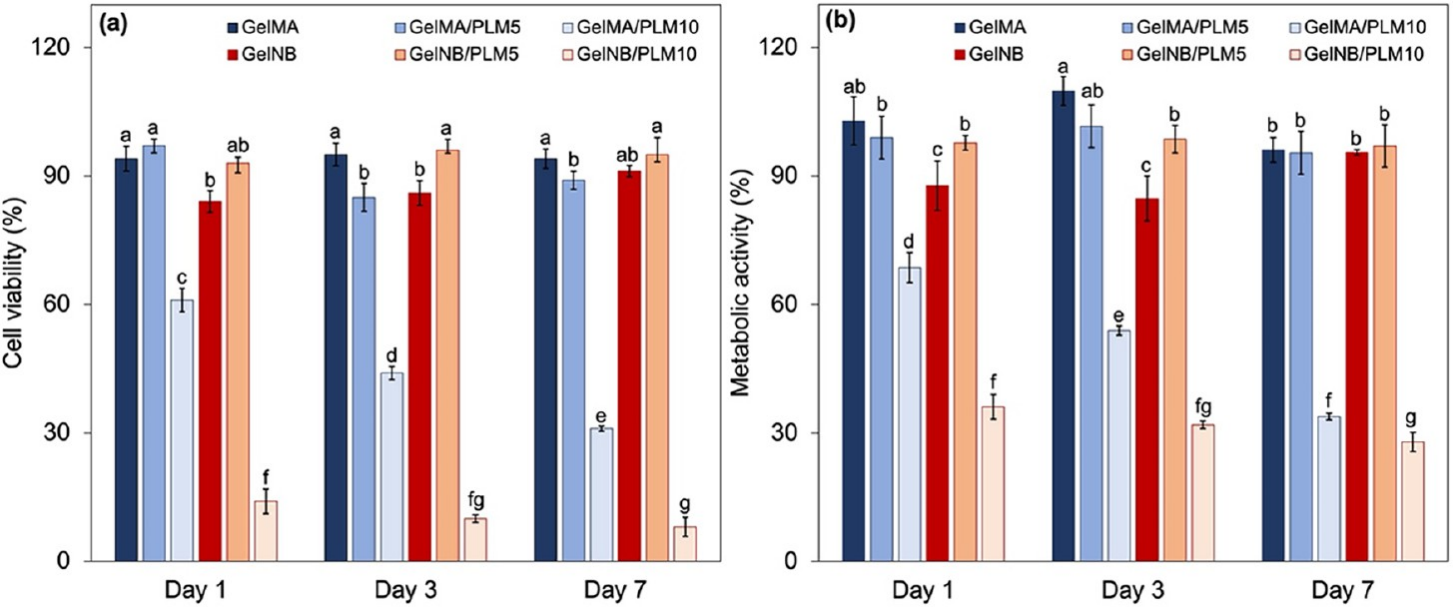
(a) quantitative live/dead data obtained after image processing using Fiji software; and (b) assessment of metabolic activity using the MTS assay. Different letters (a, b, c, d, etc.) indicate statistically different groups according to one-way ANOVA followed by Tukey’s test (*p* < 0.05).

Despite these attractive findings, an adverse impact on cell morphology was observed in the GelMA/PLM10 and GelNB/PLM10 samples [Figure 7]. This was evidenced by the presence of red dots indicative of cell death as early as the first day of contact, with morphological deterioration persisting throughout the testing period. The decline in cell viability was particularly pronounced after day 7, consistent with the results of the metabolic activity assay [Figure 8(b)]. Incorporating 10% PLM led to a notable reduction in metabolic activity, with levels dropping to approximately 30% for GelMA/PLM10 and 25% for GelNB/PLM10 after day 7. This underscores the concentration-dependent cytotoxicity of the materials, with higher PLM concentrations correlating with greater adverse effects. The heightened cytotoxicity observed in GelNB/PLM10 hydrogels, as evidenced by both live/dead staining and metabolic activity assays, could be attributed to the nearly 40% higher diffusivity (*D*) of PLM from GelNB/PLM10 compared to GelMA/PLM10 (determined previously).

A growing body of evidence indicates that the biological safety of terpenoid-derived materials is strongly influenced by molecular weight. Low-molar-mass terpene species, including monomers and short oligomers, readily interact with lipid membranes and exhibit dose-dependent cytotoxicity.^79–81^ For instance, the IC_50_ of LIM in a cytotoxicity assay using Balb/c 3T3-A31 fibroblasts after 48 h of exposure was reported to be 1.58 ± 0.26 mM (approximately 0.215 mg mL^–1^).^81^ A similar concentration-dependent behavior is expected for the limonene-based oligomer (PLM) employed in this work. In samples containing 10% PLM, an absolute amount of approximately 2 mg of oligomer was incorporated, and even though less than 25% of this mass is released, the local concentration in the pericellular region may transiently approach or exceed the IC_50_ reported for LIM, which is consistent with the marked reduction in cell viability observed for GelMA/PLM10 and GelNB/PLM10. Thus, PLM-induced cytotoxicity is governed not only by its nominal loading in the hydrogel but also by its release kinetics and diffusional behavior, which help to rationalize why the 5% (w/w) formulation remains cytocompatible, whereas the 10% (w/w) formulation reaches a concentration range associated with cytotoxic effects. Although the PLM backbone is not fully biodegradable, limonene-derived oligomers and low-molar-mass fragments are expected to follow metabolic routes analogous to LIM, involving hepatic cytochrome P450 oxidation and subsequent conjugation,^82^ thereby limiting systemic accumulation under the short-term topical exposure conditions evaluated here. Taken together, these findings suggest that 5% (w/w) represents a practical upper limit for PLM incorporation in the present hydrogel system, and that the optimal PLM content may need to be further adjusted in other matrices with distinct release profiles.

### 3.7 *In-Vivo* Wound-Healing Effects

After a comprehensive physicochemical characterization to assess the impact of PLM addition on cross-linking and the resulting hydrogel properties, GelNB/PLM5 emerged as the most promising formulation. This PLM dosage did not hinder the cross-linking process, consistent with the step growth thiol–ene photopolymerization mechanism. Moreover, it provided excellent antioxidant capacity [Figure 6(b)] while maintaining *in vitro* cytocompatibility [Figures 7 and 8(a,b)]. Consequently, GelNB/PLM5 was selected for *in vivo* biocompatibility tests to determine its safety in animal models. For comparison, hydrogels without additives (GelNB) were also analyzed, alongside a commercial advanced wound dressing (Sorbalgon) as a positive control and cotton gauze as a negative control. In total, four samples were evaluated over 18 days, with dressing changes, photographic documentation, and wound area estimation every 3 days, as illustrated in Figure 9. The excisional wound model used in this study involved the surgical removal of the epidermis, dermis, and subcutaneous fat layers, allowing for the investigation of hemorrhage, inflammation, granulation, tissue formation, reepithelialization, neovascularization, and wound remodeling.^83–85^

**Figure 9:**
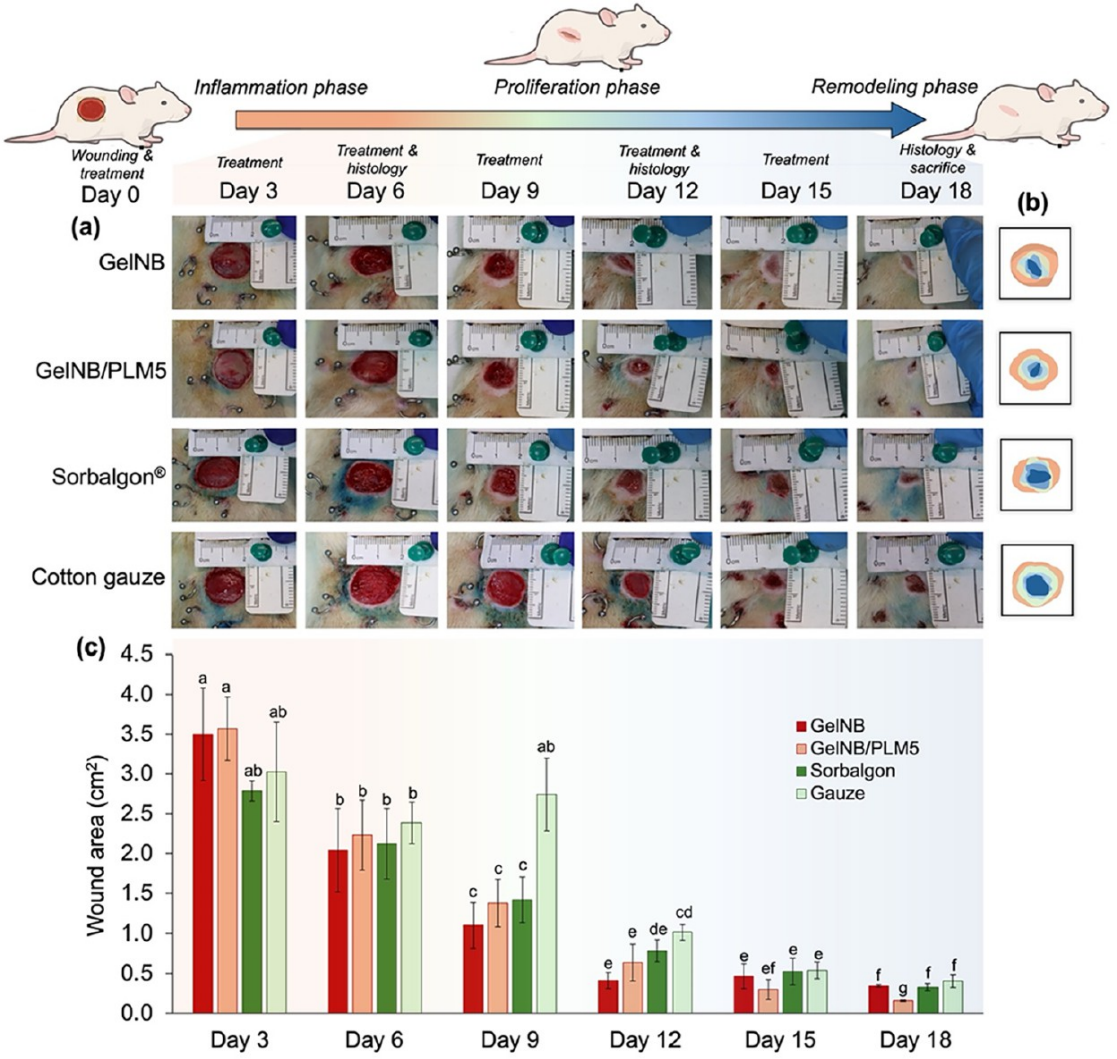
*In-vivo* wound healing study of GelNB and GelNB/PLM5-based hydrogels in comparison to traditional commercial dressings for similar applications, with (a) the photos of the wounds every 3 days during the 18-days study, (b) trace of wound closure from day 0 to day 18, and (c) the reduction in wound area during the treatment. Different letters (a, b, c, d, etc.) indicate statistically different groups according to one-way ANOVA followed by Tukey’s test (*p* < 0.05).

On day 0, wounds were standardized to a diameter of 2 cm, corresponding to an initial area of 3.14 cm^2^. The most notable observation in Figure 9(a) is the excellent biocompatibility of GelNB/PLM5, as it did not elicit an increased inflammatory response compared to GelNB in the early days, reinforcing the findings of the previous cytocompatibility analysis. On days 3 and 6, no statistically significant differences (*p* > 0.05) were observed in the mean wound areas across the four dressings [Figure 9(b,c)]. However, wounds treated with Sorbalgon exhibited a lower standard deviation and a tendency toward area reduction. Sorbalgon is a commercially available alginate-based dressing widely used in clinical practice for superficial wounds. It is particularly effective for wounds with moderate exudation, as it forms a gel upon contact with wound fluid. Additionally, it is indicated for treating leg ulcers, pressure ulcers, diabetic ulcers, burns, and surgical wounds.^86^ Although Sorbalgon exhibits faster early contraction due to its high absorbency and ionic gel formation, GelNB/PLM5 hydrogels achieve comparable healing outcomes at later stages through a bioactive mechanism.

By day 9, a significant difference was observed between the wound treated with cotton gauze and those treated with other dressings (*p* < 0.05). Wounds covered with GelNB, GelNB/PLM5, and Sorbalgon remained statistically similar on this day, but all exhibited a significant reduction in area compared to day 6. In contrast, the wound treated with cotton gauze showed no significant reduction [Figure 9(c)], indicating that this conventional dressing is less effective for this kind of wound closure, particularly in short treatment periods. Cotton gauze, a widely used wound dressing, is associated with impaired healing and scarring. It can cause tissue damage, pain, and injury during removal due to capillary disruption. Additionally, if fibers remain in the wound bed, they may contribute to granuloma formation. Cotton gauze also facilitates wound drying, which is linked to higher infection rates.^87,88^

From day 15 onward, GelNB/PLM5 displayed a trend toward enhanced wound closure, although it remained statistically similar to the other treatments (*p* > 0.05) particularly on this day. By day 18, the estimated wound closure was 88.96 ± 0.49% for GelNB, 94.90 ± 0.32% for GelNB/PLM5, 89.49 ± 1.46% for Sorbalgon, and 87.05 ± 2.55% for cotton gauze. Notably, the wound area treated with GelNB/PLM5 was significantly smaller than those treated with other dressings (*p* < 0.05), underscoring its superior efficacy in the final phase of treatment. It is well-known that gelatin plays a crucial role in promoting neovascularization, collagen fiber deposition, and follicle repair.^89–91^ The primary advantage of the GelNB/PLM5 formulation is its exceptional exudate absorption capacity, which significantly surpasses that of its GelMA-based counterpart, as demonstrated in the swelling degree studies [Figure 2(d)]. Additionally, GelNB/PLM5 exhibited remarkable *in vitro* antioxidant activity [Figure 6(b)], which may have positively contributed to its superior wound-healing performance. Antioxidants play a crucial role in the later stages of wound healing, particularly during the transition from inflammation to tissue formation and remodeling. While reactive oxygen species (ROS) are essential for initiating the healing process, excessive ROS production can lead to oxidative stress, impairing fibroblast function and neovascularization, ultimately delaying healing and increasing the risk of chronic, nonhealing wounds.^92–95^

Maintaining ROS balance is vital in the final stages of wound healing, as low levels support healing, whereas excessive ROS can induce cellular damage and apoptosis, preventing effective tissue regeneration.^92,96^ Studies indicate that antioxidants help scavenge excess ROS, mitigating their harmful effects and fostering an environment conducive to fibroblast proliferation and collagen deposition, a key processes for wound closure.^11,97^ In particular, the Nrf2/HO-1 pathway has been shown to enhance wound healing by upregulating antioxidant enzymes, thereby reducing oxidative stress and inflammation.^96^ These *in vivo* wound healing findings confirm that the PLM dosage used falls within a safe range, reinforcing its potential for future clinical applications as an innovative antioxidant additive from renewable sources.

The histological analysis corroborates the macroscopic wound closure observations, offering valuable insights into the biological processes driving tissue repair. As shown in Figure 10, wounds treated with GelNB/PLM5 exhibited markedly accelerated tissue regeneration compared to other groups. On day 6, both GelNB and GelNB/PLM5 promoted early granulation tissue formation with organized fibroblast distribution and reduced inflammatory infiltrate, contrasting with the cotton gauze group, which remained in the inflammatory phase characterized by dense neutrophilic infiltration, edema, and absence of reepithelialization. Sorbalgon treatment yielded intermediate outcomes, showing partial granulation but still considerable inflammatory presence.

**Figure 10:**
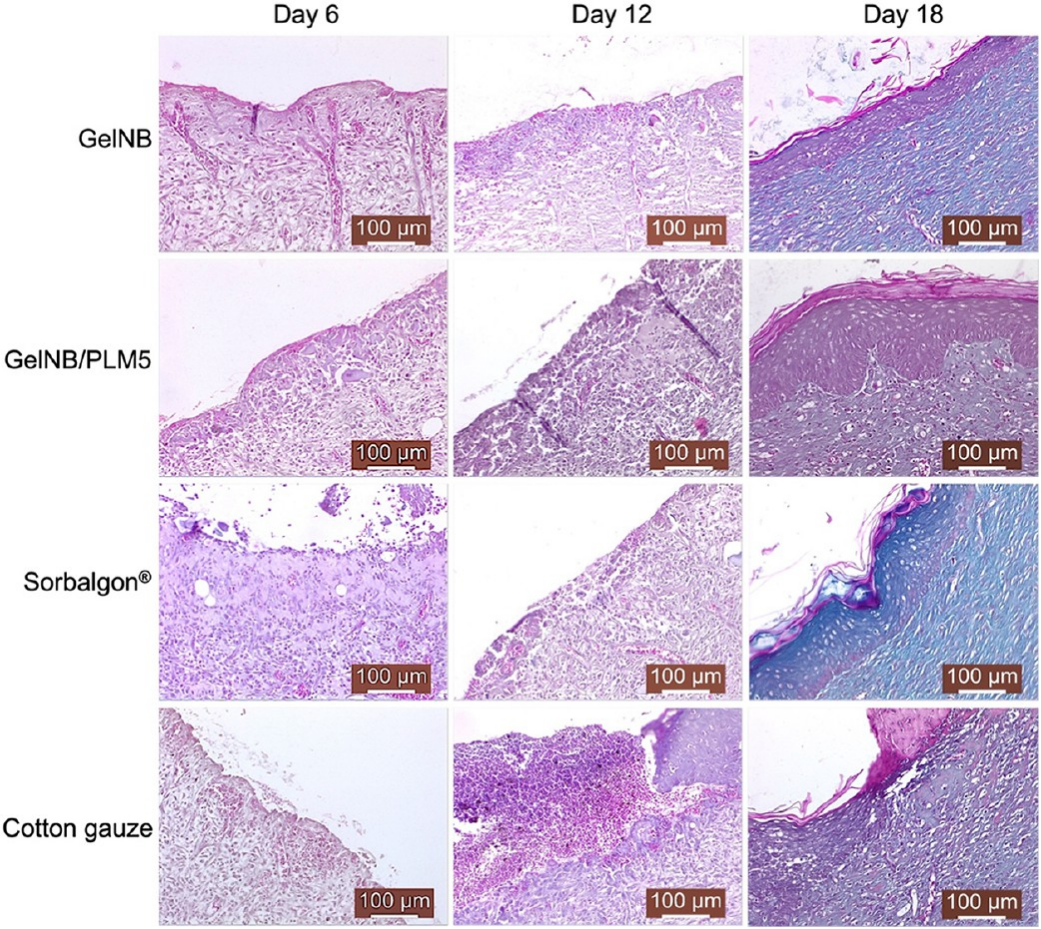
Masson’s trichrome staining of back wound tissues collected on days 6, 12, and 18 from animals treated with different wound dressings.

By day 12, the GelNB/PLM5 group demonstrated extensive reepithelialization with a continuous epidermal layer, robust fibroblast activity, and early stages of collagen deposition, indicative of a rapid transition to the proliferative phase. In comparison, the wounds treated with GelNB alone also showed substantial healing but with slightly less organized dermal architecture. Meanwhile, Sorbalgon maintained moderate granulation and partial reepithelialization, whereas the cotton gauze group showed persistent disorganization, high cellular debris, and poor epithelial closure, consistent with delayed healing dynamics. On day 18, the differences became even more pronounced. GelNB/PLM5-treated wounds displayed a fully regenerated stratified epithelium with well-formed rete ridges and a dense, highly organized collagen network, as confirmed by Masson’s trichrome staining. The dermis exhibited minimal residual inflammation, suggesting the wound had entered the remodeling phase with mature tissue architecture. GelNB-treated wounds achieved comparable epidermal restoration but exhibited slightly looser collagen networks, indicating that the antioxidant activity provided by PLM further enhanced matrix maturation. Sorbalgon-treated wounds, although fully closed, presented thinner epithelium and less dense collagen, characteristic of a less advanced remodeling stage. In contrast, the cotton gauze group showed incomplete epidermal restoration and poorly organized extracellular matrix, strongly indicating a risk of chronic wound formation and fibrosis.^92,93^

Overall, these histological results reinforce the macroscopic observations, confirming that GelNB/PLM5 not only accelerates wound closure but also enhances the quality of tissue regeneration. This superior outcome is attributed to a synergistic combination of the favorable physicochemical properties of the hydrogel matrix and the antioxidant effects of PLM, which collectively may have mitigated oxidative stress.^92^ Moreover, the absence of any histological signs of cytotoxicity or foreign body reaction further supports the biocompatibility of GelNB/PLM5, validating its potential as a sustainable, multifunctional wound dressing with clinical relevance.

## 4 Conclusions

A comparative analysis of GelMA and GelNB hydrogels incorporating PLM prior to cross-linking reveals that differences in photo-cross-linking mechanisms critically influence the resulting networks’ physicochemical, structural, and functional properties. In GelMA, chain-growth polymerization was susceptible to radical scavenging by PLM, significantly reducing both cross-linking density and double bond conversion. In contrast, GelNB hydrogels, synthesized via thiol–ene step-growth polymerization, retained structural regularity and high double bond conversion despite the presence of PLM. These distinctions result in markedly different physicochemical and release behaviors: GelNB matrices exhibited more efficient PLM diffusion, attributed to their higher swelling capacity and more uniform network architecture. All formulations followed a burst-to-sustained release profile governed by Fickian diffusion. Antioxidant assays confirmed intrinsic radical scavenging activity in both systems, with GelNB demonstrating superior efficacy. Incorporation of 5% (w/w) PLM (GelNB/PLM5%) significantly enhanced antioxidant capacity, with DPPH radical scavenging increasing by over 80%, suggesting a potential synergistic effect between the norbornene-functionalized matrix and the PLM component. *In-vitro* assays confirmed that GelNB/PLM5% formulation supported adequate fibroblast viability, while *in vivo* wound healing studies demonstrated favorable biocompatibility, with unobserved inflammatory response in the beginning and increased wound closures in the final stage. Collectively, these findings highlight that GelNB is more attractive than GelMA when antioxidants must be incorporated before cross-linking. Particularly, in this study, GelNB/PLM5% is considered the most promising formulation for future applications, offering a combination of structural performance, sustained release profile, strong antioxidant effect, and biological safety.

## Supplementary Material



## Notes

Detailed synthetic procedures, NMR characterization of GelMA, GelNB, and PLM, the determination of functionalization degrees, and kinetic parameters of PLM release (PDF)
