# Supplementary material for: Bioactive Limonene-Derived Oligomer in Gelatin Hydrogels: Impact of Cross-Linking Chemistry on Physicochemical Properties and Wound Healing Performance
Source: ACS Appl Mater Interfaces. 2025 Dec 24;18(1):629–48. doi: 10.1021/acsami.5c20409 (PMC12781062; doi:10.1021/acsami.5c20409)
Supplement: Supplementary file 1 [file am5c20409_si_001.pdf]

Supporting Information

Bioactive limonene-derived oligomer in gelatin hydrogels: impact of crosslinking chemistry on physico-chemical properties and wound healing performance

Roniérik Pioli Vieira,[a,*] Guilherme Frey Schutz,[a] Laurens Parmentier,[b] Oana-Maria Chirliu,[c] Aurelian-Sorin Pasca,[d] Lenuta Profire,[c] and Sandra Van Vlierberghe[b,*]

[a] Universidade Estadual de Campinas (UNICAMP), School of Chemical Engineering (FEQ), Albert Einstein Avenue, 500, 13083-852 Campinas, São Paulo, Brazil. [*]Email: ronierik@unicamp.br

[b] Polymer Chemistry and Biomaterials Group (PBM), Centre of Macromolecular Chemistry (CMaC), Department of Organic and Macromolecular Chemistry, Faculty of Sciences, Ghent University, Krijgslaan 281, S4, 9000 Ghent, Belgium. [*]Email: Sandra.VanVlierberghe@UGent.be

[c] Department of Pharmaceutical and Therapeutic Chemistry, Faculty of Pharmacy, "Grigore T. Popa" University of Medicine and Pharmacy of Iași, 16 University Street, 700115 Iași, Romania.

[d] Faculty of Veterinary Medicine, "Ion Ionescu de la Brad" University of Life Sciences, 8 Mihail Sadoveanu Alley, 700489 Iași, Romania.

Synthesis of gelatin methacrylolyl (GelMA) and limonene oligomer (PLM)

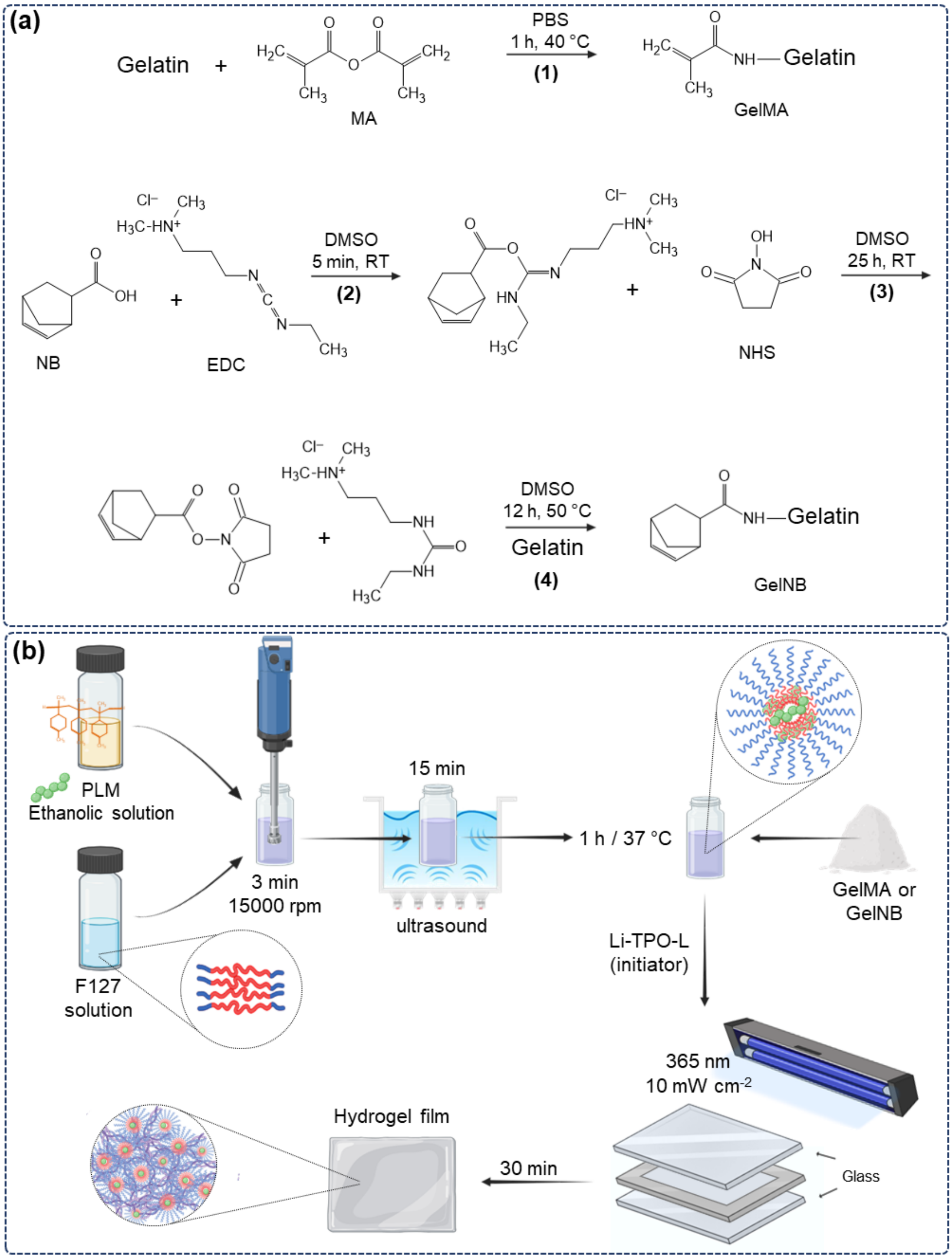

Figure S1. Schematic overview of the synthesis of (a) GelMA through the direct reaction of gelatin and methacrylic anhydride (MA) (reaction 1), and GelNB (reaction 4) with preceding activation of 5-norbornene-2-carboxylic acid (NB) with 1-ethyl-3-(3-dimethylaminopropyl)carbodiimide (EDC) (reaction 2), followed by reaction with N-

hydroxysuccinimide (NHS) (reaction 3) to obtain the reactive succinimidyl ester; and (b) simplified illustration to obtain hydrogel films containing PLM emulsions.

^{1}H NMR spectroscopy was employed to determine the amount of methacrylamide in GelMA. Figure S1(a) illustrates that in comparison with the ^{1}H NMR spectrum of gelatin, new peaks related to the methacryloyl moieties appeared in the GelMA spectrum, at 5.7 and 5.5 ppm (blue highlight), for protons [CH_2=C(CH_3)CONH–] of the incorporated methacrylamide groups, and at 2.1 ppm for methyl protons [CH_2=C(CH_3)CO–] of the same groups. Additionally, the free lysine protons signal at 3.2 ppm ($NH_2CH_2CH_2CH_2CH_2$–) of the unmodified gelatin practically disappeared in the spectrum of GelMA, confirming its high conversion in methacrylamide moieties.

The degree of substitution (DS) in GelMA was determined by utilizing the signal at 1.0 ppm, which corresponds to the resonance of the methylene protons in valine (Val), leucine (Leu), and isoleucine (Ile) side chains. These alkyl side chains are considered inert, serving as a suitable reference. In gelatin type B the total number of protons from the valine, leucine, and isoleucine side chains amounts to 18 (6 protons in Val, 6 protons in Leu, and 6 protons in Ile), equivalent to 0.386 mol/100 g of gelatin. Additionally, the total amount of primary amines available for the reaction is 0.0385 mol of NH_2 (lysine)/100 g of gelatin. The values of the integral corresponding to the protons of the introduced methacrylamide (at 5.5 and 5.7 ppm, i.e., $I_{5.5} + I_{5.7} = 3.45$) and the integral of the reference signal (i.e., $I_{1.0} = 18$) were inserted in Equation S1 and a DS ≈ 95.6% was obtained. The ^{1}H NMR data were acquired using a Bruker WH 500 MHz instrument at 40 °C, with D_2O employed as the solvent.

$$DS(\%) = 0.384 \times \left(\frac{\frac{I_{5.5}+I_{5.7}}{2}}{I_{1.0}} \right) mol \times \frac{100}{0.0385\, mol\, NH_2} \quad \text{(Eq. S1)}$$

Similarly, the DS for the gelatin functionalized with 5-norbornene 2-carboxylic acid was obtained from the “ene” functionality present in the norbornene groups that appeared in the ^{1}H NMR spectrum of GelNB, Figure S1(a). First, the integral of the reference signal at 1.0 ppm was calibrated as 18 protons (reference). Then, the integral of the norbornene signals was determined. It is important to note that the norbornene-derived gelatin retains a mixture of the two isomers of 5-norbornene 2-carboxylic acid,

leading to the observation of four distinct signals at approximately 6.06, 6.33, 6.34, and 6.39 ppm. The summation of their respective integrals amounted to 3.059. Upon incorporating these integral values into Equation S2, a DS ≈ 84.8% was obtained.

$$DS(\%) = 0.384 \times \left(\frac{\frac{I_{6.06}+I_{6.32}+I_{6.35}+I_{6.39}}{2}}{I_{1.0}} \right) mol \times \frac{100}{0.0385\ mol\ NH_2} \quad \text{(Eq. S2)}$$

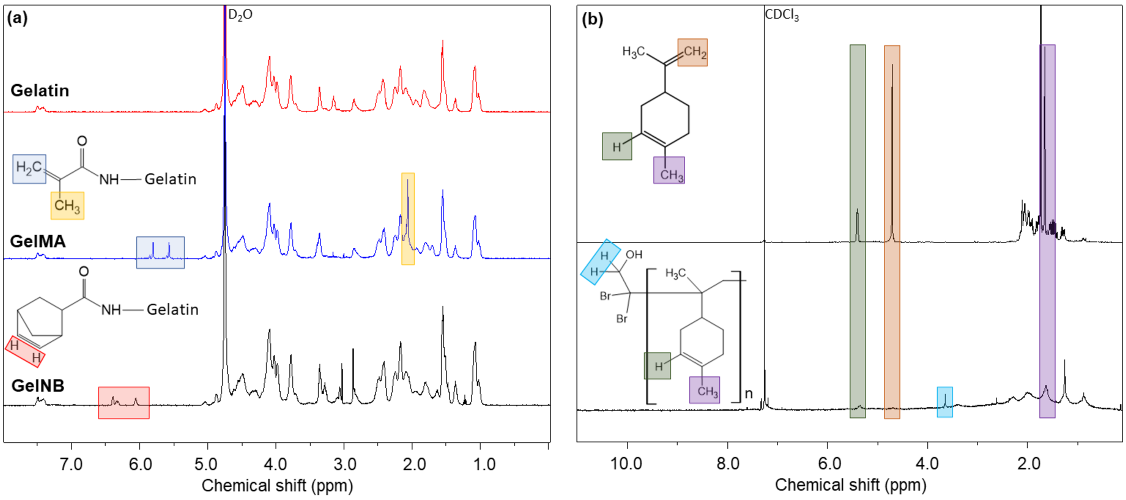

Figure S2. (a) ^{1}H NMR spectra of gelatin, GelMA, and GelNB, with the highlighted main signals used for DS determination; and (b) ^{1}H NMR spectra of limonene (LIM) and the derived oligomer (PLM) synthesized previously, which was reproduced with permission from Elsevier, Copyright®, 2025.[1]

The $\overline{M}_n$ of polylimonene (PLM) was determined using Equation S3, considering the integration of initiator protons derived from the ^{1}H NMR spectrum. The NMR spectrum was obtained using a Bruker 300 MHz instrument at 25 °C, with $CDCl_3$ employed as the solvent. To facilitate accurate analysis, the peak at 3.65 ppm was used as reference peak. Subsequently, the integrals of the other peaks were normalized using the known number of initiator protons per PLM molecule as reference.

$$\overline{M}_n = MW_{initiator} + n \times MW_{monomer} \quad \text{(Eq. S3)}$$

in which $MW_{initiator}$ = 282.78 g mol^{-1} for 2,2,2-tribromoethanol, $MW_{monomer}$ = 136.24 g mol^{-1}, and n ≈ 5.

To estimate the degree of polymerization (n), we considered the sum of integrals corresponding to the protons in saturated carbons within the range of 0.5 to 2.5 ppm. This sum encompasses the total number of protons in a repeating unit of PLM. The

degree of polymerization was then calculated by dividing the total number of protons in this specified region by the number of saturated protons in a repeating unit (i.e., 15), yielding n ≈ 5, which means an $\overline{M}_n$ ≈ 964 Da, considering that only 2,2,2-tribromoethanol acted as initiator.

Table S1. Determined kinetic parameters for PLM release from gelatin hydrogels.

Sample	Peppas-Sahlin	Weibull
**GelMA/PLM5**	k_1 = -26.981	f = 18.090
	k_2 = 33.899	a = 2.120
	n = 0.056	b = 0.449
	WSSR = 0.054	WSSR = 0.049
**GelMA/PLM10**	k_1 = 10.122	f = 21.586
	k_2 = -1.253	a = 1.848
	n = 0.519	b = 0.627
	WSSR = 0.143	WSSR = 0.041
**AlgNB/PLM5**	k_1 = -70.049	f = 19.36
	k_2 = 78.997	a = 1.647
	n = 0.031	b = 0.450
	WSSR = 0.136	WSSR = 0.049
**GelNB/PLM10**	k_1 = 13.643	f = 23.234
	k_2 = -2.109	a = 1.4227
	n = 0.438	b = 0.542
	WSSR = 0.121	WSSR = 0.055

References

Vieira, R. P.; Schutz, G. F.; Parmentier, L.; Van Vlierberghe, S. Effect of Polylimonene on the Release Behavior and Physico-Chemical Properties of Photo-Cross-Linkable Alginate-Based Hydrogels. *Sustainable Materials and Technologies* **2025**, *43*, e01311. https://doi.org/https://doi.org/10.1016/j.susmat.2025.e01311.
